# Surface engineering of ZnCl_2_-activated N-doped water hyacinth hydrochar: a mechanistic insight for fast and efficient removal of Cr(vi) and crystal violet dye

**DOI:** 10.1039/d6ra07071j

**Published:** 2026-09-08

**Authors:** Md. Hassan Shahriar, Nahida Akter, Md. Tanvir Shahriar, Tasfia Jaman Ahana, Md. Mahadi Hasan, Md. Rezwan Miah, S. M. Nizam Uddin

**Affiliations:** a Department of Chemistry, Graduate School of Physical Sciences, Shahjalal University of Science and Technology Sylhet-3114 Bangladesh; b Department of Chemistry, University of Barishal Barishal-8254 Bangladesh sohel-che@sust.edu nakter@bu.ac.bd

## Abstract

In this study, ZnCl_2_-activated nitrogen-doped water hyacinth (*Eichhornia crassipes*) hydrochar (Ac-NWHHC) was synthesized from water hyacinth *via* a two-step hydrothermal method. The purpose was to develop a high-performance activated hydrochar using a flexible and efficient synthesis strategy for the remediation of toxic organic dye (Crystal Violet) and heavy metal Cr(vi). The XPS analysis of the Ac-NWHHC revealed the successful N incorporation, which existed as pyrrolic, pyridinic, and graphitic-N forms in the hydrochar matrix. BET analysis showed that the hydrothermal activation process led to an enhanced porous structure, expanding the material's overall active surface area. The Ac-NWHHC removed approximately 85% of crystal violet (CV) dye and 82% of Cr(vi) using 0.5 g L^−1^ adsorbent from their synthetic wastewater. The adsorption processes display better resemblance to the Freundlich isotherm, suggesting multilayer adsorption nature on the heterogeneous surface. The maximum adsorption capacity of Ac-NWHHC was found to be 42.63 mg g^−1^ for crystal violet and 113.12 mg g^−1^ for Cr(vi) species. Kinetic analysis indicated both adsorption processes followed the pseudo-second-order kinetic model. Both the adsorption processes were found to be thermodynamically spontaneous and of endothermic nature due to the negative Gibb's free energy (Δ*G*°) changes and positive values of enthalpy changes (Δ*H*) Therefore, it can be concluded that Ac-NWHHC can demonstrate substantial adsorption for effluent treatment due to its low-cost precursor, facile synthesis method, and excellent adsorption performance.

## Abstract 2

Surface-engineered hydrochar from water hyacinth biomass enables rapid and high-capacity removal of Cr(vi) and crystal violet dye *via* synergistic complexation, reduction and π–π and/or H-bonding interactions.

## Graphical abstract

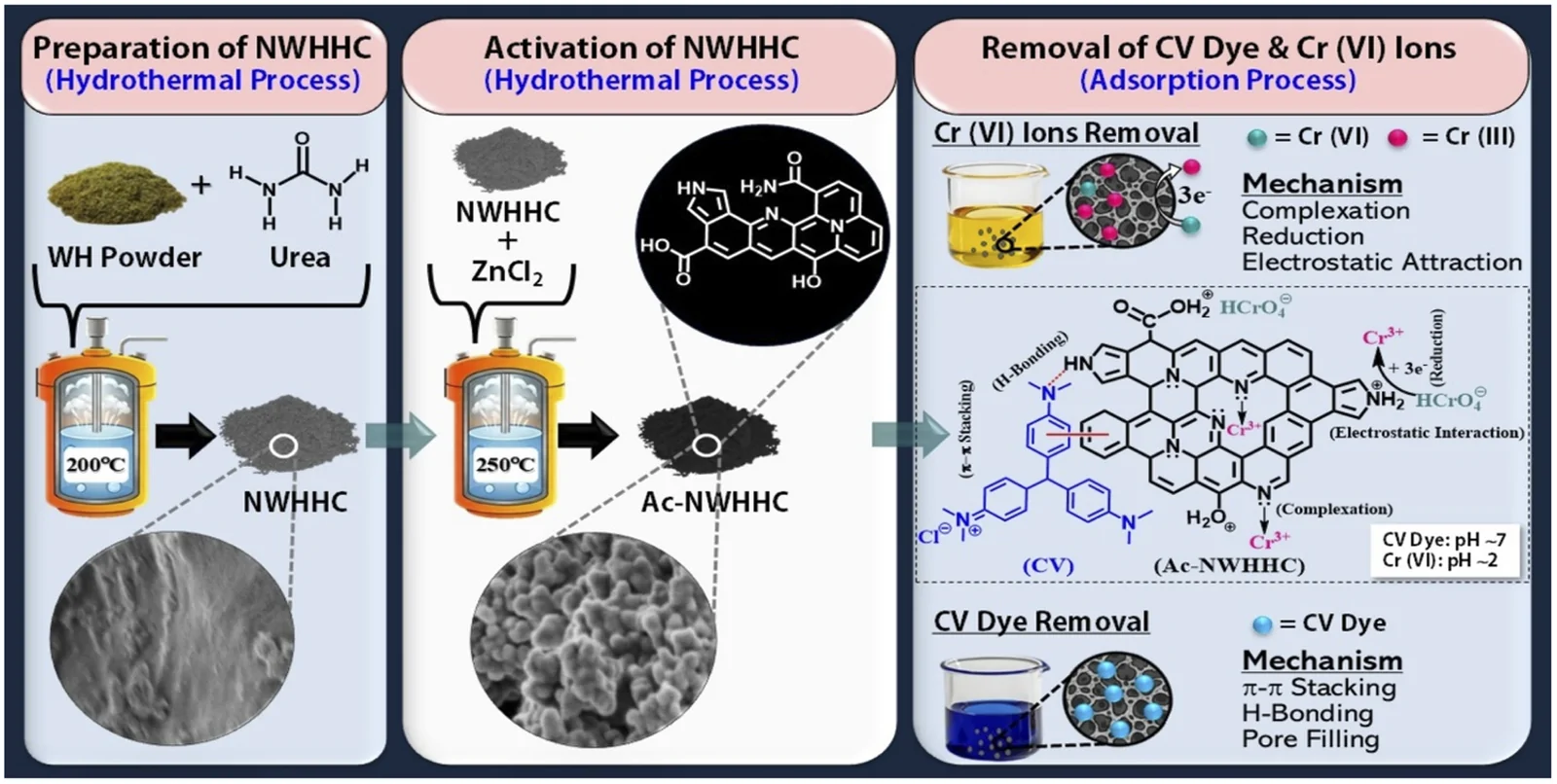

## 1. Introduction

Globally, the supply of fresh drinking water is limited, and the effects of climate change are becoming more pronounced day by day.^1^ Rapid industrial growth, particularly in the textile, leather, cosmetics, food technology, paper, and printing sectors, releases large volumes of wastewater contaminated with organic dyes, alkalis, salts, grease, oil, and heavy metals.^2,3^ The majority of the industries in developing countries are in proximity to water resources, mostly rivers. These effluents from industries are discharged directly prior to any treatment. The main pollutants in these effluents are dyes and pigments, which have mutagenic and carcinogenic properties and seriously harm aquatic life, human health, and the food chain.^4,5^ These dyes are hazardous, frequently non-biodegradable, and constitute a great threat to ecosystems due to the reduction of dissolved oxygen and photosynthetic action in water streams by color effluent discharge.^6^ In addition, heavy metal ions (HMIs) originate from anthropogenic activities and urbanization at a very high rate. Among all, As, Hg, Cr, Pb, and Cd are considered the most hazardous heavy metals even at very low concentrations.^7,8^ According to the World Health Organization (WHO), the acceptable limits of contamination for As, Hg, Cr, Pb, and Cd ions are 0.05, 0.001, 0.05, 0.05, and 0.005 mg L^−1^, respectively.^9^ These heavy metals are ubiquitously distributed through the unplanned and unregulated use of agrochemicals and untreated industrial waste, which contaminates water and soil.^10^ Intensive intake of food and water contaminated with HMIs causes a variety of newly invented diseases. HMIs have a high bond formation ability with the active site of proteins or enzymes that alter the natural biochemical life cycle of the living cell.^11,12^ HMIs are also responsible for cancers, skin lesions, cardiovascular diseases, kidney failure, and many other diseases.^13,14^ Therefore, the efficient elimination of organic dyes and HMIs from wastewater has been a hot issue for many years. Chemical precipitation, membrane filtration, floatation, electrochemical reduction, reverse osmosis, and adsorption are some of the widely utilized traditional wastewater treatment techniques.^15–18^ Though all of these methods can achieve high contaminant removal efficiencies, adsorption offers a simpler, low-energy, and cost-effective route compared to the rest. It works well across a wide range of pollutants even at trace levels, requires minimal infrastructure, is regenerable, and most importantly, scalable.^19,20^ Hence, adsorption is one of the most extensively studied strategies by scholars for eliminating both heavy metal ions and recalcitrant organic dyes from contaminated effluents. A myriad of adsorbents have been reported by scholars, including activated carbon, biochar, hydrochar, MOF (metal organic framework), COF (covalent organic framework), zeolites, metal oxides, *etc.* Carbonaceous materials derived from biomass sources have received notable focus for their utilization as adsorbents since carbon-rich materials are the most abundant on Earth.^21–25^ Hydrochar is one of the value-added products obtained from the hydrothermal carbonization of carbon sources. Due to milder synthesis conditions, the possibility of higher yield, and maximum retention of functionalities from the raw biomass, hydrochars have emerged as a prominent adsorbent in recent times. Even though the hydrochars can be directly utilized as adsorbent species, their efficiency can be enhanced through modifications of the hydrochar matrix *via* physical and chemical activation, surface functionalization, and heteroatom doping.^26–28^ Heteroatom incorporation introduces binding sites by altering the surface polarity in the hydrochar matrix, which has been proven to be an effective strategy in elevating the adsorption activity of the biomass-derived carbon.^29–32^ Liang *et al.* synthesized N-doped spherical carbons from glucose and urea to treat Cr(vi) contaminated water. The doped N atoms acted as active electron donors in the reduction of Cr(vi) to Cr(iii).^33^ Zhang *et al.* used wheat straw and 3-aminopropyl triethoxysilane to prepare N-functionalized hydrochar for treating both organic dyes and heavy metals. The N-rich hydrochar interacted with the adsorbates *via* π–π interactions, H-bonding, and complex formation.^34^ N-doped microporous carbons were also synthesized by Hou *et al.* from bamboo shoots to remove Methyl Orange and Rhodamine B dyes, which exhibited enhanced dye uptake capacity compared to the pristine hydrochar.^35^ Activation after carbonization is the widely followed strategy in the case of producing biomass-derived carbons with high surface area, which is a key factor governing adsorption. The most common route for hydrochar activation is treating either the raw biomass or the hydrochar with an activating agent, like KOH, H_3_PO_4_, ZnCl_2_, *etc.*, followed by a thermal treatment, called pyrolysis. Hou *et al.* reported that activation of N-doped carbon spheres with KOH exceptionally increases the material's surface area and porosity. In this activation process, the nitrogen content of N-doped carbon spheres was decreased significantly as they employed a high temperature ranging from 600 to 800 °C.^36^ Similar responses were also observed by Qu *et al.* for activating lotus stalk-derived N-doped hydrochar with KHCO_3_ at 800 °C, exhibiting enhanced Cr(vi) and Bisphenol A removal performance.^37^ V. Kitenge and his group reported a single-step pyrolysis technique for concurrently N-doping and chemical activation of tondolo peel powder with K_2_CO_3_ and urea at 700 °C under Ar flow.^38^ Thus, pyrolysis is an effective method for fabricating activated carbons to enhance the material's surface area. But it requires complex and costly instrumentation to maintain high temperature (typically 600 to 900 °C) under an inert atmosphere. Conversely, the hydrothermal method is a less complex and less energy-demanding strategy compared to the pyrolysis method. As the operating conditions of the hydrothermal method are less severe, it can also be effective in preserving the functional groups in the hydrochar even after the activation stage. Therefore, biomass-derived carbon-based material resources can be converted to a myriad of materials depending on the sources as well as the conversion processes.

Water hyacinth (*Eichhornia crassipes*) plants are highly abundant all over the world, especially in Southeast Asia, Africa, North and Central America, and Oceania. The quick growth, high cellulose content, and widespread availability of water hyacinth, an invasive aquatic species, make it a good feedstock for the manufacture of hydrochar. Even though a few successful research works have been done for wastewater treatment using water hyacinth biochars, water hyacinth hydrochars are still less studied, which requires a thorough application-based investigation.^39–42^ To the best of our knowledge, no research has yet been reported on the use of chemically activated N-doped water hyacinth hydrochar for organic dye and heavy metal remediation. Therefore, the main objectives of this work were to synthesize N-doped hydrochar from water hyacinth, followed by chemical activation with ZnCl_2_ by using a two-step hydrothermal method. To explore the potential of the prepared activated N-doped hydrochar (Ac-NWHHC) for the removal of crystal violet (CV) dye and Cr(vi) ions, a comparative study was carried out with unactivated N-doped hydrochar (NWHHC). In this work, the adsorption performance of Ac-NWHHC, kinetics, and thermodynamics were investigated for CV dye and Cr(vi) ions. In addition, the probable changes in the functional groups and surface properties in the activation steps, as well as the adsorption mechanism of the target contaminants on the adsorbent, have been explored by using advanced characterization techniques. This work offers a new alternative adsorbent for cationic CV dyes and Cr(vi) ions, utilizing a simple and effective hydrothermal process.

## 2. Experimental section

### 2.1 Materials and chemicals

The water hyacinth (*Eichhornia crassipes*) was sourced from the local ponds and lakes in Sylhet, Bangladesh. The leaves and petioles of the water hyacinths were utilized, which were thoroughly washed with Milli-Q water and ethanol. The fragments were first air-dried and then dried in a vacuum oven. Finally, the dried biomass was ground into a fine powder. All the reagents used in this work were of analytical grade and utilized as received without further purification. Urea (CH_4_N_2_O, 99.5%), anhydrous zinc chloride (ZnCl_2,_ 99.99%), crystal violet (C_25_H_30_ClN_3_), and potassium dichromate (K_2_Cr_2_O_7_, ≥99%) were purchased from Merck, Germany.

### 2.2 Synthesis of N-doped hydrochar

The powdered water hyacinth was dissolved in a urea solution, maintaining a 1 : 2 (wt/wt) ratio. The mixture was stirred for 1 hour prior to being transferred into a Teflon-lined autoclave reactor for hydrothermal treatment for 4 hours at 200 °C in an oven. A brown-colored hydrochar was obtained after the completion of hydrothermal carbonization. The hydrochar was collected through centrifugation and finally washed with Milli-Q water several times to remove any unwanted materials. At last, the hydrochar was dried in a vacuum oven overnight at 80 °C and stored in a sealed container, labeled as NWHHC.

### 2.3 Activation of N-doped hydrochar

**Fig. 1:**
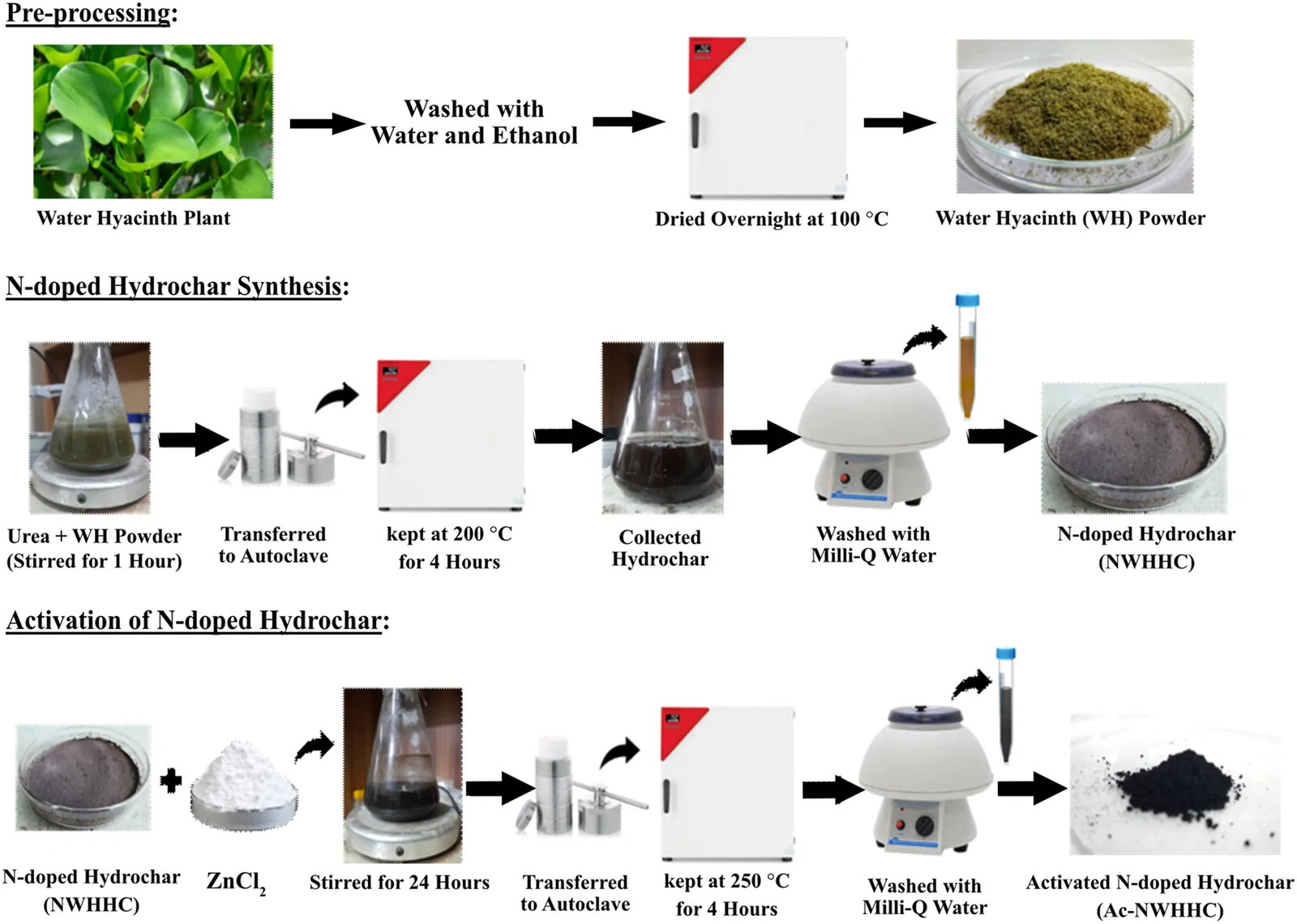
Visual illustration of Ac-NWHHC preparation from water hyacinth.

The synthesized hydrochar was mixed with ZnCl_2_ in a 1 : 3 (wt/wt) ratio in Milli-Q water and agitated for 24 hours at room temperature to achieve complete impregnation of ZnCl_2_ into the N-doped hydrochar matrix. The obtained homogeneous mixture was transferred to a Teflon-lined autoclave and then again subjected to hydrothermal treatment for 4 hours at 250 °C. The product was collected through centrifugation and first washed with 0.1 M HCl, followed by Milli-Q water until a neutral pH was achieved. The washed product was dried in a vacuum oven overnight at 80 °C. The dried product was stored and labeled as Ac-NWHHC. The schematic illustration of the step-by-step synthesis of Ac-NWHHC is shown in Fig. 1.

### 2.4 Characterization

X-ray photoelectron spectroscopy (XPS) was used to investigate the surface characteristics of the NWHHC and Ac-NWHHC using a K-Alpha XPS instrument (Thermo Fisher Scientific, USA). To study the functional groups of the samples, Fourier-transform infrared (FTIR) spectroscopy was used in the ATR mode using a 4800S spectrometer (Shimadzu, Japan). A field emission scanning electron microscopy (FE-SEM) coupled with energy dispersive X-ray spectrometry (EDS) was used to explore the surface morphology and elemental composition of the NWHHC and Ac-NWHHC by using a Thermo Fisher Scientific Apreo 2S Lo-Vac SEM instrument. Surface area and porosity of the prepared materials were analyzed by using a high-performance gas adsorption analyzer ASAP 2020 Plus Version 2.0 (Micromeritics, USA). A thermogravimetric analysis was performed using the TGA-50 (Shimadzu, Japan).

### 2.5 Batch adsorption of crystal violet (CV) dye and Cr(vi) ions

An investigation of the effects of several parameters, including contact time, pH, adsorbate concentration, adsorbent dose, and co-existing ions, were conducted for both Crystal Violet (CV) dye and Cr(vi) ions. The adsorbate solutions of desired concentrations in each of the batch experiments were prepared by diluting stock solutions of 100 mg L^−1^ for each adsorbate. To study the effect of chemical activation on sorption performance, a contact time study was conducted for both NWHHC and Ac-NWHHC at various time intervals. For each investigated contact time, the adsorbent was added to 10 mL of the adsorbate solution at a dose of 0.5 g L^−1^ in individual experimental runs for the different time frames. This similar approach was utilized for the other subsequent parameter investigations. A Shimadzu UV-1900i UV-Vis Spectrophotometer was used to quantify the changes in CV dye concentration, whereas a Shimadzu AA-7000 Atomic Absorption Spectrophotometer was used to track the total Cr [Cr(vi) and Cr(iii)] concentration changes across all studied parameters. During each investigation, the supernatant was separated from the adsorbent–adsorbate mixture by centrifugation and then subjected to spectral analysis. From the spectral quantitative analysis, the % DR (dye removal efficiency), % MR (metal removal efficiency) and *q*_*t*_ (adsorption capacity, mg g^−1^) of Ac-NWHHC were estimated using the following equations:1
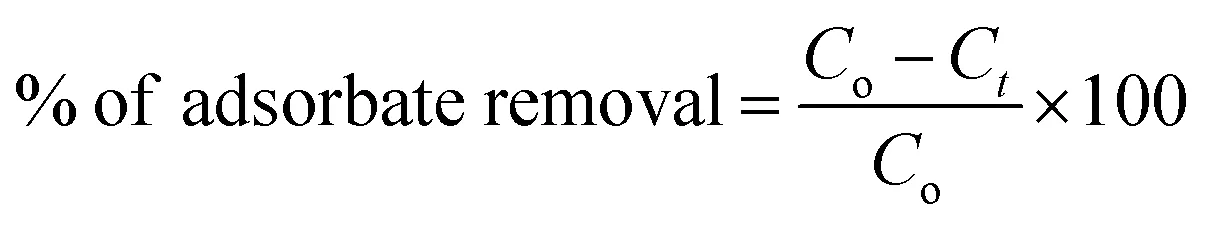
2
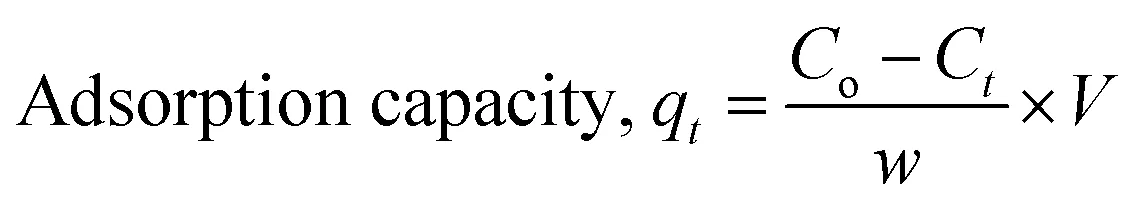
Here, *C*_o_ (mg L^−1^) and *C*_*t*_ (mg L^−1^) represent the adsorbate concentrations at time *t* = 0 and *t* = *t* minute, respectively. *q*_*t*_ (mg g^−1^) is the mass of adsorbate adsorbed per gram of the adsorbent. *w* (g) is the mass of the added adsorbent, and *V* (L) expresses the volume of adsorbate taken in each investigation.

### 2.6 Adsorption kinetics studies

Kinetic studies are crucial for elucidating the key aspects of an adsorption process, including the rate of adsorbate uptake, the underlying mechanisms that govern adsorption, and the nature of the adsorption. Pseudo-first-order (PFO), pseudo-second-order (PSO), Elovich, and Weber–Morris (W–M) models were applied to study the kinetics associated with the adsorption processes. The kinetic parameters were evaluated using the following equations:^43–45^

Pseudo 1st order kinetic equation:

3 ln(*q*_e_ − *q*_*t*_) = ln *q*_e_ − *k*_1_*t*

Pseudo 2nd order kinetic equation:4
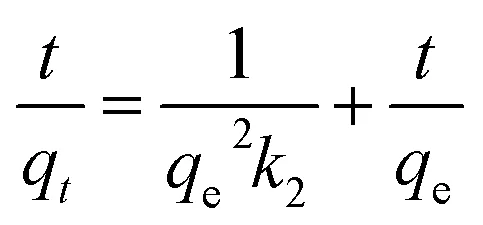


Elovich kinetic equation:5
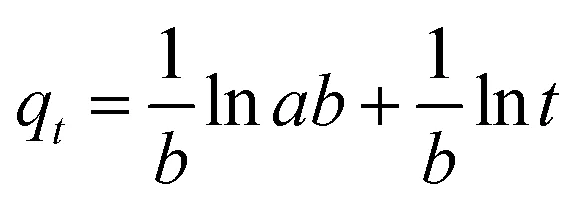


Weber–Morris kinetic equation:

6 *q*_*t*_ = *k*_W−M_*t*^0.5^

In the above equations, *q*_e_ (mg g^−1^) and *q*_*t*_ (mg g^−1^) represent the mass (mg) of CV dye or Cr(vi) adsorbed per gram of the adsorbent at the equilibrium time, and at time *t*, respectively. *k*_1_ (min^−1^) and *k*_2_ (g mg^−1^ min^−1^) are the rate constants of the adsorption process following the PFO and the PSO rate laws, respectively. *a* (mg g^−1^ min^−1^) is the initial sorption rate, and *b* (g mg^−1^) is related to the rate of desorption and is a function of surface coverage and activation energy. *k*_W–M_ (mg g^−1^ min^−0.5^) represents the Weber–Morris rate constant.

### 2.7 Adsorption isotherm studies

To determine the adsorption capacity and gain insight into the mechanism associated with the adsorption processes, the Langmuir, the Freundlich, and the Temkin isotherms were studied. Although the classical density functional theory (cDFT) is an effective approach for describing the adsorption properties of a functionalized material and addressing the interactions of the adsorbate at the solid–fluid interface, its application requires detailed system-specific information, as well as substantial computational resources.^46,47^ Therefore, the widely adopted empirical isotherm models were employed to analyze the experimental data and correlate the adsorption behavior of CV dye and Cr(vi) onto Ac-NWHHC. The parameters associated with the studies were derived using the following equations:^48^

Langmuir adsorption isotherm equation:7
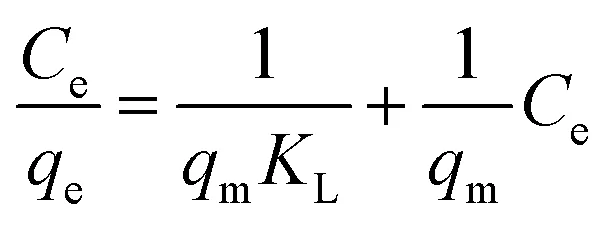


Freundlich adsorption isotherm equation:8
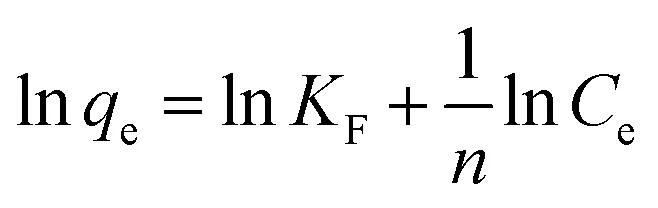


Temkin adsorption isotherm equation:

9 *q*_e_ = *B*_T_ ln *K*_T_ + *B*_T_ ln C

In the above equations, *C*_e_ (mg L^−1^) and *C*_o_ (mg L^−1^) represent the equilibrium and the initial concentrations of the CV dye and Cr(vi), respectively. *q*_m_ (mg g^−1^) is the maximum adsorption capacity of the adsorbent predicted by the Langmuir isotherm. *K*_L_ (L mg^−1^) is the Langmuir constant, which expresses the adsorption capacity, and *K*_F_ (L mg^−1^) and are the Freundlich constants, which express adsorption capacity and adsorption intensity, respectively. *B*_T_ is the Temkin isotherm constant, and it is associated with the heat of adsorption. *K*_T_ (L mg^−1^) is the Temkin equilibrium constant.

## 3. Results and discussion

### 3.1 Materials characterization

**Fig. 2:**
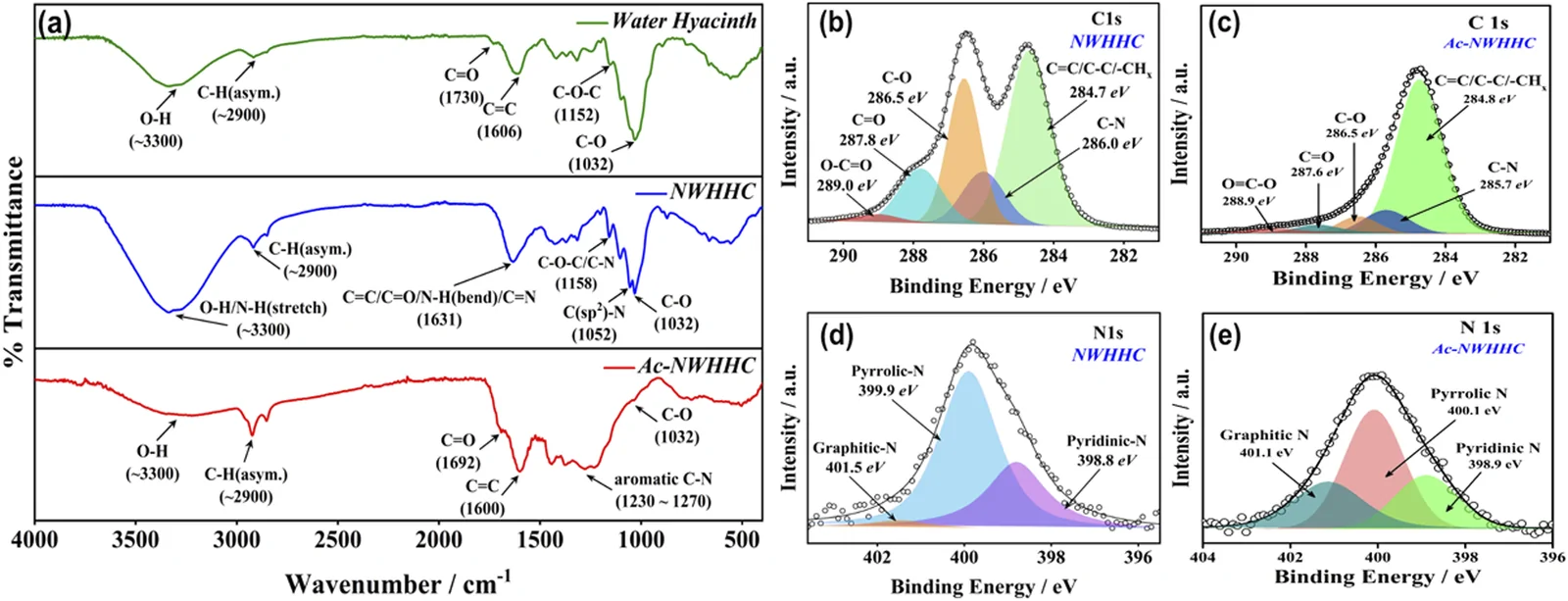
(a) The FTIR spectra of the raw biomass, NWHHC, and Ac-NWHHC. The deconvoluted C 1s XPS spectra of (b) NWHHC and (c) Ac-NWHHC. The deconvoluted N 1s XPS spectra of (d) NWHHC and (e) Ac-NWHHC.

The functional groups of the water hyacinth powder, NWHHC, and Ac-NWHHC were investigated by FTIR as shown in Fig. 2a. In the FTIR spectrum of the water hyacinth, the prominent bands observed at about 1032, 1152, 1606, 1730, 2900, and 3300 cm^−1^ originated from the stretching of the glycosidic C–O–H and C–O–C bonds in cellulose, aromatic C=C in lignin, C=O in hemicellulose, C–H in methylene, and O–H in the major hemicellulose and cellulose, respectively.^49–53^ In NWHHC, a noticeable sharp increase was observed in the intensity of the broad O–H stretching band, whereas a significant decrease was observed in that of Ac-NWHHC. This increased intensity could result from an additional contribution of the N–H stretching vibration originating from the amine or amide groups formed in the NWHHC matrix, while the reduced intensity in Ac-NWHHC could be attributed to the enhanced dehydration and aromatization of the N-doped hydrochar in the presence of ZnCl_2_ at the higher activation temperature.^54,55^ The shifting of the broad peak from about 1606 to 1631 cm^−1^ in NWHHC could have contributions from the aromatic C=C, the C=O, the C=N stretches, and the N–H in-plane bending.^56,57^ When activated, a lower shift to ∼1600 cm^−1^, along with a shoulder at ∼1692 cm^−1^, was noticed. This observation can be ascribed to the dominance of the conjugated aromatic feature upon further carbonization of NWHHC, along with slight retention of the C=O functional groups.^58^ The peak at 1152 cm^−1^ in the raw biomass also shifted to a higher wavenumber of 1158 cm^−1^ in NWHHC, attributed to the contributions from the C–N stretching band of the amines.^34^ The shifting of the C–O peak into that of C–N indicated the incorporation of N into the hydrochar matrix. The sharp adjacent peak that appeared at 1052 cm^−1^ in NWHHC is ascribed to the C(sp^2^)-N stretching vibration.^37,59^ The decreased intensity of the C–O stretching bands in Ac-NWHHC in the range of 1030–1100 cm^−1^ indicates that the degree of oxygen functionalities in NWHHC decreased after activation. Finally, the intense overlapped bands that appeared in the range of 1230–1276 cm^−1^ in the spectrum of Ac-NWHHC originated from the C–N stretch in the conjugated aromatic matrix of the hydrochar upon activation. The band could be attributed to the newly stabilized N-functionalities such as the pyrrolic-, pyridinic-, and graphitic-N moieties. In addition, XPS analysis was executed to further investigate the functional groups of the samples. The deconvoluted C 1s high-resolution spectra of both NWHHC and Ac-NWHHC in Fig. 2b and c displayed five peaks at ∼284.8, ∼285.8, ∼286.6, 287.8, and ∼289.0 eV corresponding to C=C/C–C/–CH_*x*_, C–N, C–O, C=O, and O–C=O groups, respectively.^37,60–64^ The low-intensity peaks of C–O, C=O, and O–C=O groups were observed in Ac-NWHHC compared to NWHHC, indicating that the degree of oxygen functionalities decreased after activation, which is relevant to the observation of the high-resolution O 1s XPS spectra (Fig. S1) and the FTIR analysis mentioned above. The presence of the N 1s peak in both spectra confirms the successful incorporation of nitrogen into the hydrochar matrix and retention after the activation process. The deconvoluted N 1s high-resolution spectra of NWHHC and Ac-NWHHC in Fig. 2d and e showed three peaks at around ∼400.1, ∼398.9, and ∼401.1 eV for pyrrolic or amino-N, pyridinic-N, and graphitic-N, respectively.^34,62,65^

**Fig. 3:**
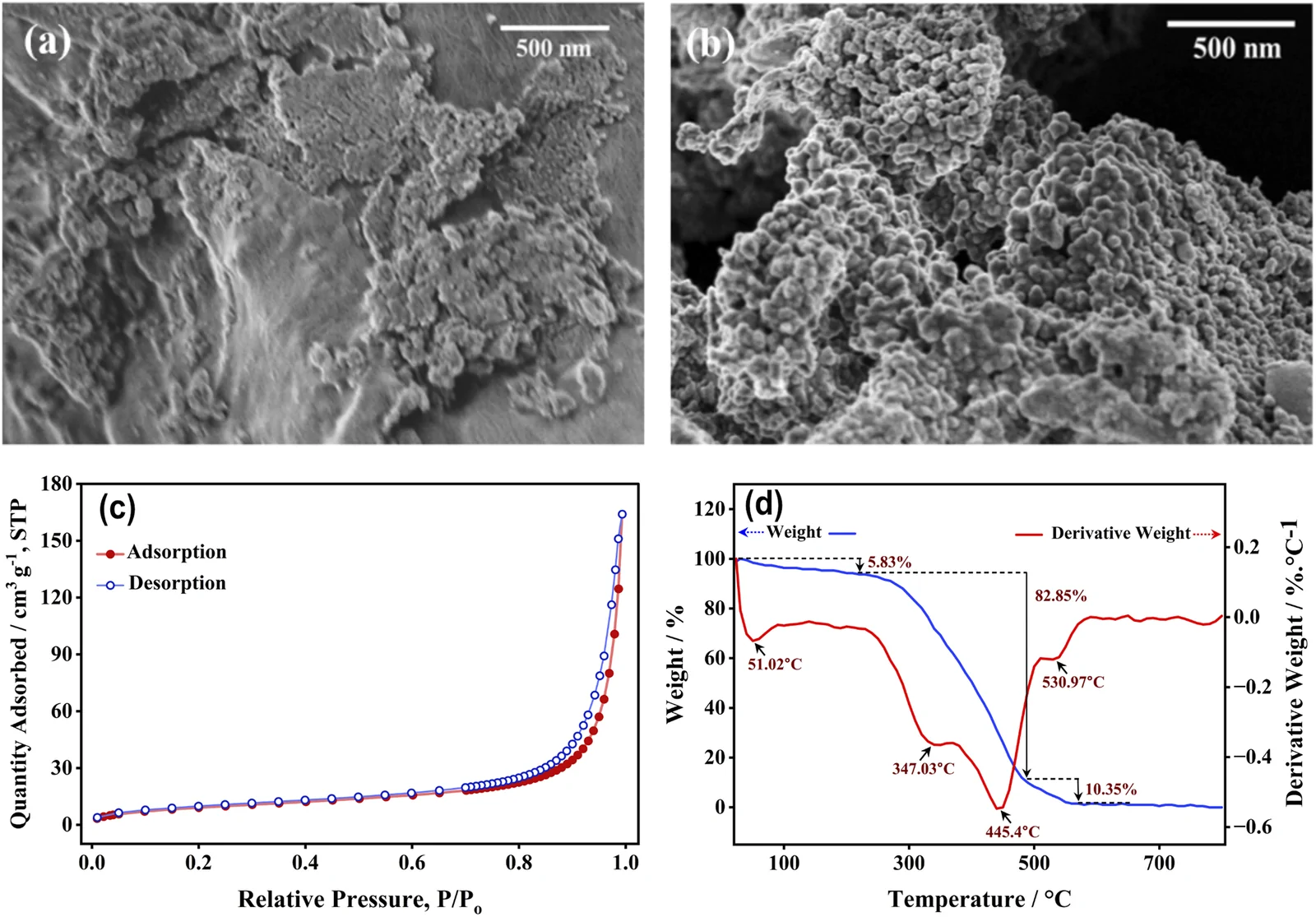
FESEM images of (a) NWHHC & (b) Ac-NWHHC, (c) N_2_ adsorption–desorption isotherm of Ac-NWHHC at 77 K, and (d) TGA-DTG profile of Ac-NWHHC.

During the hydrothermal carbonization, alongside the thermal conversion (hydrolysis, dehydration, decarboxylation, condensation, polymerization, *etc.*) of the raw biomass, *etc.*, urea decomposed into NH_3_ and isocyanic acid (HNCO), with the latter further decomposing to NH_3_ and CO_2_.^66^ Then, the –NH_2_ groups underwent the Maillard and Mannich-type reactions with the furfurals, 5-hydroxymethylfurfurals (HMF), and the phenolic fragments, which were formed during the thermal degradation of the lignocellulosic components of the raw biomass, and incorporated the various types of nitrogenous functional groups (amino-N, amidic-N, pyrrolic-N, pyridinic-N, *etc.*) within the carbon matrix in NWHHC.^67–69^ The increased intensity of the C=C peak in Ac-NWHHC [Fig. 2c] implies a higher degree of carbonization and aromatization of the carbon matrix of NWHHC during the activation step. The impregnation of ZnCl_2_ into the hydrochar matrix led to the breakdown of more macromolecules in the NWHHC, such as the unchanged lignin fragments.^70,71^ ZnCl_2_ accelerated the rate of dehydration and decarboxylation of those thermally stable fragments (–OH, –C=O) at the elevated activation temperature by reacting with the H_2_O molecules generated from the elimination of these functional groups.^58^ Even after such chemical activation, an appreciable amount of oxygen functionalities was retained in the Ac-NWHHC. The majority of the incorporated nitrogen in the NWHHC was retained post-activation, showing the effectiveness of the hydrothermal activation method. In Fig. 2e, the binding energy of the Pyrrolic-N moieties shifted from 399.9 eV in NWHHC to 400.2 eV in Ac-NWHHC, which could be attributed to the conversion of the amino and amide groups in NWHHC to a more stable form, like pyrrolic-N, *via* dehydration and deamination during the activation stage.^67,69^ Hence, the binding energy (400.2 eV) is more suggestive of pyrrolic-N in Ac-NWHHC. After activation, the content of graphitic-N in Ac-NWHHC increased, which can be observed from the increase in the relative peak area of the graphitic-N in Ac-NWHHC [Fig. 2e]. The decrease in the relative peak area of pyridinic-N indicates its conversion to the more stable graphitic-N form.^72^ The surface morphology and elemental composition of the synthesized materials were investigated using FE-SEM and EDS. Fig. 3a and b illustrates the FE-SEM images of NWHHC and Ac-NWHHC, demonstrating a significant morphological change in NWHHC after activation. In Fig. 3a, the NWHHC showed a relatively sheet-like or aggregated curved bulk morphology. As NWHHC was activated, the morphology changed to quasi-spherical granules with large inter-granular voids [Fig. 3b]. The granules were mainly composed of carbon decorated with N and O functionalities, as revealed by the EDS elemental analysis (Fig. S2). The granules had an average particle size of ∼60 nm, as estimated by measuring the diameter of the particles from the SEM images. The formation of these fine nanosized particles was attributed to the stabilization of the particle surface in the hypersaline environment created by the high concentration of ZnCl_2_ in the activation mixture.^73^ The carbon fragments, formed due to the rapid dehydration and decarboxylation induced by ZnCl_2_, were stabilized by the high ionic strength of the reaction medium, preventing their further growth and suppressing Ostwald ripening.^74,75^ This effectively drove dehydration from the inner matrix of the hydrochar structure, and the acid-washing after the hydrothermal treatment removed the products, which eventually led to exposure of the interparticular gaps.

Surface area and porosity of Ac-NWHHC were also analyzed by using a high-performance gas adsorption analyzer. As shown in Fig. 3c, the N_2_ adsorption–desorption isotherm of Ac-NWHHC is of type-IV(a) with a hysteresis at the higher relative pressure region according to the International Union of Pure and Applied Chemistry (IUPAC). The corresponding type of isotherm is generally associated with materials having wider mesopores (2–50 nm) than normal.^76^ The N_2_ intake was slow at lower relative pressure, but as the pressure increased, N_2_ adsorption increased with an H3-type hysteresis loop, without any limiting adsorption at the higher end.^77^ The absence of a steeper increase in N_2_ volume at low relative pressure and a sharp increase in that at higher relative pressure refer to the lack of micropores and dominance of mesopores in the Ac-NWHHC, respectively.^69,78^ However, the BET surface area, *S*_BET_ of Ac-NWHHC calculated from the linear region in the isotherm plot was 34.8 m^2^ g^−1^, while that for the NWHHC was only 4.5 m^2^ g^−1^ (Fig. S3). The enhanced value of the *S*_BET_ of the Ac-NWHHC highlighted the significant expansion of the surface area upon chemical activation, also indicating the success of the hydrothermal activation method.

Finally, the thermal decomposition profile of Ac-NWHHC was explored by TGA and DTG analysis, as shown in Fig. 3d. It presents both the temperature-dependent mass loss behavior as well as the rate of mass loss as a function of temperature. The DTG profile exhibited three distinct stages of thermal degradation of Ac-NWHHC. The first stage of mass loss was very slight and took place within the range of ∼150 °C, with the maximum loss at ∼50 °C,^39^ which is attributed to the evaporation of physically adsorbed water on the surface or trapped inside the hydrochar structure. The absence of any mass loss in the DTG curve in the temperature range of 200–300 °C indicates the absence of any hemicellulose portion in Ac-NWHHC.^79^ The TGA curve became steeper in the second stage, revealing a substantial mass loss of ∼83% in the temperature range of 250–500 °C, with the rate of mass loss peaking at ∼445 °C with a shoulder at ∼347 °C. The pronounced decrease in mass within this temperature interval reflects the expulsion of the majority of the volatile components from Ac-NWHHC.^64^ The small shoulder peak obtained at 347 °C could have a contribution from the decomposition of cellulose residues,^80^ and thermally labile functional groups, like the carbonyl and carboxyl groups. The highest mass loss obtained at ∼445 °C is attributed to the pyrolysis of more recalcitrant structures, like lignin residues.^69,71^ The minimal resistance of Ac-NWHHC to thermal degradation could be attributed to the inverse relation of the enhanced mesoporosity of the activated hydrochar to thermal resistance.^81,82^ In the third stage, the TGA curve reached a plateau, signaling no significant mass loss. The minor mass loss peak at ∼530 °C in DTG could correspond to the decomposition of highly stable N-functional groups (pyrrolic-N).^83^

### 3.2 Adsorption studies

#### 3.2.1 Effect of adsorbate solution pH

**Fig. 4:**
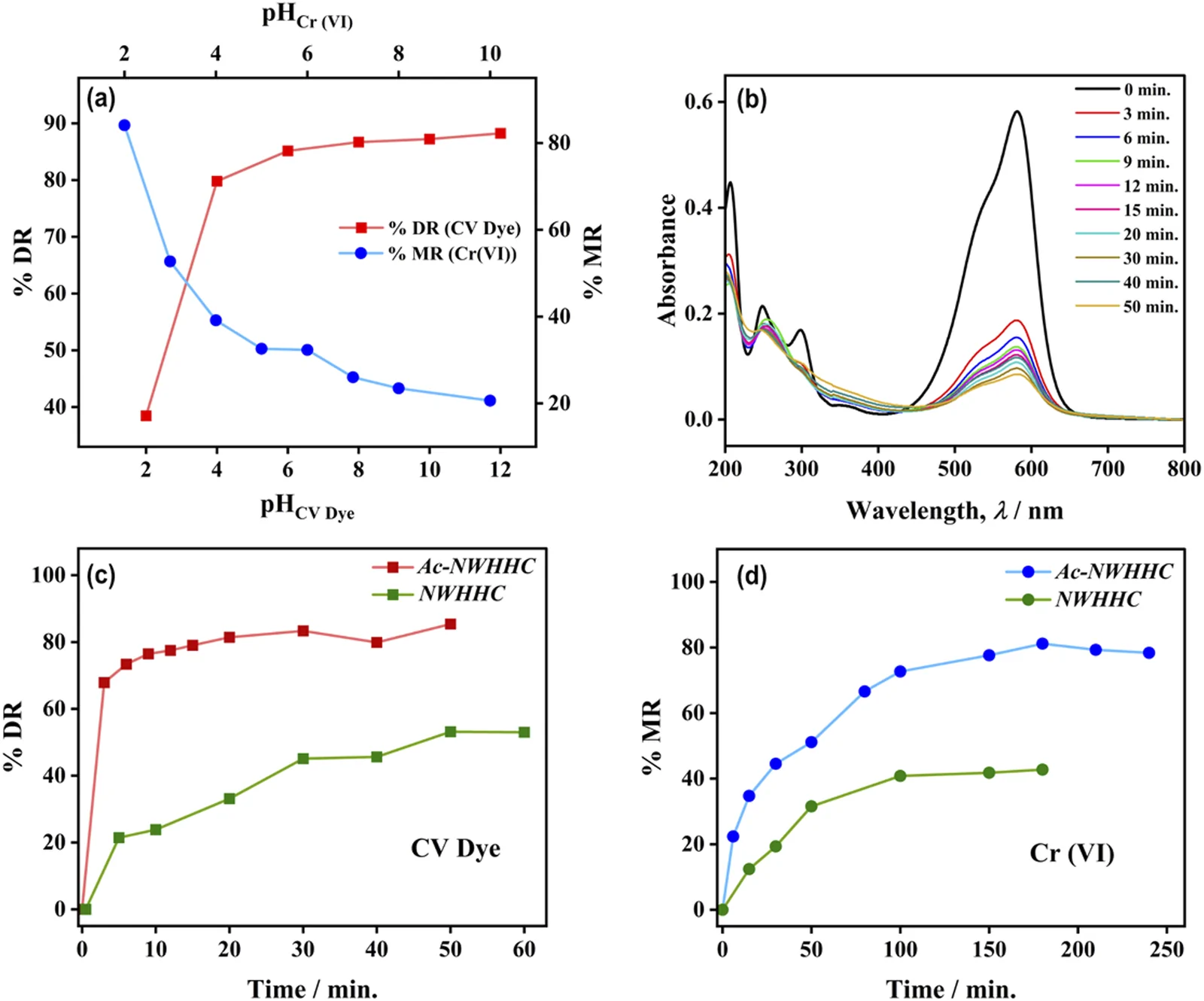
(a) Effect of solution pH on the removal of CV dye and Cr(vi), (b) temporal changes of UV-Vis. Spectra of CV dye with time using Ac-NWHHC, and the effect of contact time on % of (c) CV dye and (d) Cr(vi) removal by Ac-NWHHC and NWHHC. The experimental parameters were *C*_o_ = 10 mg L^−1^ (CV dye) and 30 mg L^−1^ (Cr(vi)), adsorbent dose = 0.5 g L^−1^, volume = 10 mL (in each run), pH = 6.3 (CV dye) and 2.0 (Cr(vi)), and temperature = room temperature.

The effect of pH on CV dye and Cr(vi) removal was investigated and shown in Fig. 4a, where a gradual and steady increase in the % DR of CV and a decrease in the % MR of Cr(vi) were observed with increasing pH. The variations of CV dye and Cr(vi) uptake can be explained by considering the changes in surface charges with the pH of the point of zero charge (pH_pzc_) of the adsorbent. The Ac-NWHHC surface was found to have a net zero charge at pH_pzc_ ∼5.1 (Fig. S4). It illustrates that at a pH < pH_pzc_, the surface of the adsorbent becomes positive due to the protonation of the surface functional groups. Whereas at pH > pH_pzc_, the surface becomes negative as the functional groups lose protons. Therefore, adsorption of CV dye onto Ac-NWHHC can be resisted by the strong electrostatic repulsion between the protonated functional groups and the cationic CV dye molecules at a highly acidic pH, hence reducing the % DR value. However, at pH 4.0, a significant increase in the percentage of CV dye removal was observed, and beyond this pH, the removal efficiency further increased with subsequent pH increments and remained almost consistent. This increase in the rate of CV removal below the pH_pzc_ can be attributed to the subordination of electrostatic interactions and the dominance of other non-electrostatic interactions, such as the saturation of the mesopores, as well as phenomena like π–π stacking interactions, n–π type donor–acceptor interactions, hydrogen bonding, *etc.*^34,84^

At higher pH beyond pH_pzc_, the deprotonation of the surface functional groups on Ac-NWHHC led to increased electrostatic interaction and became an additional contributory factor in enhancing the adsorption of the cationic CV dye.^85^ Though Ac-NWHHC exhibited a remarkable removal of the CV dye in a broad range of pH, it exhibited the highest Cr(vi) removal at pH 2.0, which can be attributed to the presence of the Cr(vi) ions as HCrO_4_^−^ and Cr_2_O_7_^2−^ species at the acidic pH range of the precursor solution, with HCrO_4_^−^ being the dominant species at pH 2.0. The surface positive charge of Ac-NWHHC at pH < pH_pzc_ resulted in an enhanced attraction of the negatively charged HCrO_4_^−^ anions on the adsorbent surface.^33^ As the solution became more acidic, the HCrO_4_^−^ content increased in the solution. Moreover, the abundance of the H^+^ ions in the solution at lower pH facilitated the reduction of the adsorbed Cr(vi) species.^37^ At pH > 6.0, the Cr(vi) ions existed in the CrO_4_^2−^ form, and the surface negative charge at pH > 5.1 led to decreased Cr(vi) uptake owing to the repulsive electrostatic interaction between the anionic Cr(vi) species and the surface.^86^ As Ac-NWHHC showed a significantly high and consistent % DR of CV dye over a broad range of pH, the subsequent investigations for CV dye were conducted at the pH of the as-prepared dye solution, which was around 6.3; and as for the Cr(vi) sorption investigation, the rest of the parameters were studied at the optimum pH of 2.0.

#### 3.2.2 Effect of contact time

Investigations of contact time were conducted for the adsorption of CV dye and Cr(vi) onto Ac-NWHHC and NWHHC. Fig. 4b shows the UV-Vis responses of CV dye removal on Ac-NWHHC with respect to time, and the comparative efficiency of Ac-NWHHC and NWHHC in removing CV dye and Cr(vi) are shown in Fig. 4c and d, respectively. Using an adsorbent dose of 0.5 g L^−1^, the Ac-NWHHC adsorbed ∼85.4% CV dye and ∼81.16% Cr(vi) from their precursor solutions, whereas NWHHC removed ∼53.17% CV dye and ∼47.74% Cr(vi) under similar conditions, revealing the higher absorptivity of Ac-NWHHC over NWHHC. The superior adsorption efficiency of the Ac-NWHHC can be explained by the conversion of the NWHHC into quasi-spherical nanosized granules upon activation, which possess enhanced surface area compared to the inactivated NWHHC with the flat sheet-like morphology [Fig. 2a and b]. The wide inter-granular voids in Ac-NWHHC due to the chemical activation also contributed to the enhancement of surface area, exposing more area for the adsorbate species. Moreover, the N-functionalities in Ac-NWHHC acted as additional active sites of adsorption along with the O-functional groups, providing sites for binding and interaction for the CV dye molecules and the Cr(vi) species with the Ac-NWHHC surface. The % DR of CV dye was found to be the steepest in the first 5 minutes, followed by a sluggish adsorption rate, and then reached equilibrium at about 15 minutes. It indicates that the initial CV dye adsorption rate was dominated by instantaneous diffusion of dye onto the exterior surface and the mesoporous vacant sites of the adsorbent. In the case of Cr(vi), there was a gradual increase in % MR of Cr(vi) at the first 40–60 minutes, which later slowed down to proceed towards the equilibrium. The initial gradual adsorption of Cr(vi) may be ascribed to the predominant diffusion of the HCrO_4_^−^ species through the mesopores of the adsorbent because of their smaller size. However, the rate of the CV dye uptake was quite rapid, whereas a steady pace of removal was observed for the Cr(vi) species. The slower uptake of Cr(vi) compared to the CV dye could be due to the “adsorption-reduction” mechanism associated with Cr(vi) removal by carbon-based materials, which could lead to delayed removal of Cr(vi).^87,88^

#### 3.2.3 Effect of Ac-NWHHC dosage

**Fig. 5:**
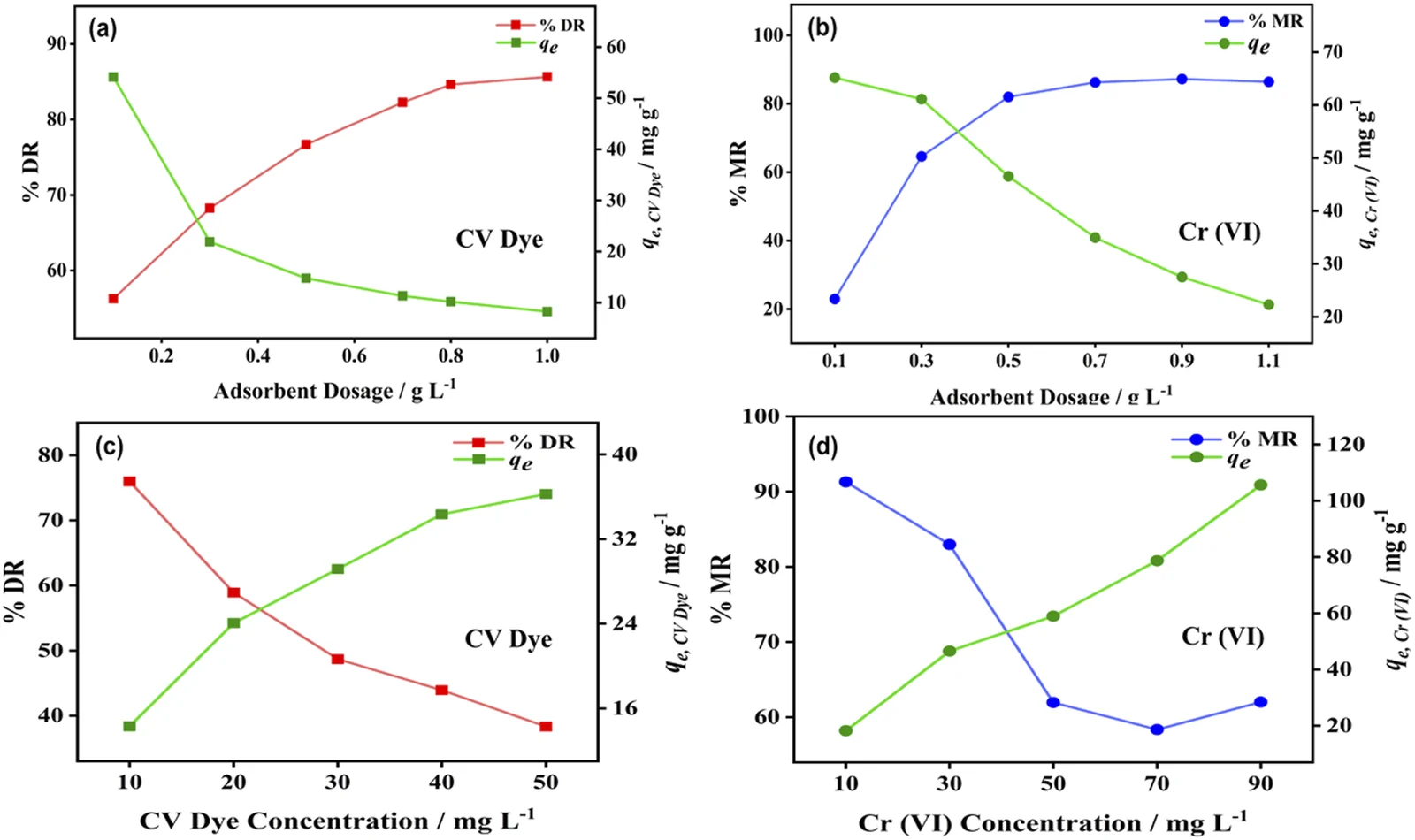
Effect of Ac-NWHHC dosage (a) CV dye and (b) Cr(vi). Effect of initial concentration of (c) CV dye and (d) Cr(vi) on the adsorption efficiency of Ac-NWHHC at room temperature. The experimental parameters were pH = 6.3 (CV) and 2.0 (Cr(vi)), *C*_o_ = 10 mg L^−1^ (CV) and 30 mg L^−1^ (Cr(vi)) for adsorbent dose effect, adsorbent dose = 0.5 g L^−1^ for concentration effect, contact time = 10 min. (CV) and 300 min. (Cr(vi)), volume = 10 mL.

Fig. 5a and b represent the accelerating trend in the removal percentage of CV dye and Cr(vi) along with the Ac-NWHHC dosage increment. Ac-NWHHC was added to the adsorbate solutions within the range of 0.1–1 g L^−1^, maintaining a constant concentration, which resulted in an increase from 56.25 to 85.64% CV dye removal and 22.96 to 86.42% Cr(vi) adsorption. These ascending patterns of adsorbate removal with the increase of adsorbent dosages can be explained by the increased surface area and active sites with the increments of the adsorbent dosages. The increased surface area and vacant active sites led to a higher degree of surface interactions between the adsorbate species and adsorbent, enhancing the %DR of CV dye and %MR of Cr(vi).^6,89,90^ However, the percentage of CV and Cr(vi) removal didn't increase significantly after a dosage of 0.7 g L^−1^ and 0.6 g L^−1^ of Ac-NWHHC, respectively. The plateau after a certain dosage was ascribed to the establishment of the equilibrium stage in the sorption system. Even though the CV and Cr(vi) removal efficiency increased with the increasing Ac-NWHHC dosage, the adsorbate uptake capacity (*q*_*e*_) decreased. This inverse relationship arises from the incomplete utilization of the sorption sites on Ac-NWHHC, since the fixed quantity of adsorbate molecules became distributed across a larger number of active sites.^91^

#### 3.2.4 Effect of adsorbate concentration

As evident from Fig. 5c and d, the percentage of removal for both adsorbate species followed a decreasing trend with the increasing concentration levels of the corresponding precursor solutions. The percentage of CV dye removal decreased from 76% to 38.34% as the concentration increased from 10 to 50 mg L^−1^, and the Cr(vi) removal percentage decreased from 91.3% to 60.7% with a Cr(vi) concentration increment of 10 to 90 mg L^−1^. This declining trend is attributed to the progressive saturation of the sorption sites, as the number of CV dye molecules and HCrO_4_^−^ species in solution increasingly exceeded the number of accessible active sites. A second contributing factor could be the increased repulsive force exerted by the adsorbed CV dye molecules and the HCrO_4_^−^ anions on the unadsorbed species as those approached the active sites for binding.^92^ Higher initial adsorbate concentration in the system led to a corresponding enhancement in the uptake capacity, *q*_*e*_, of Ac-NWHHC. For CV dye [Fig. 5c], it went from 14.31 to 36.26 mg g^−1^, while an increase in capacity from 18.26 to 105.62 mg g^−1^ of Cr(vi) [Fig. 5d] was observed.

This increment in the uptake was attributed to the greater number of dye molecules and HCrO_4_^−^ ions at higher concentrations, which enhanced the concentration gradient between the bulk solution and the Ac-NWHHC surface and increased the rate of diffusion of the adsorbate species into the adsorbent. As a result, the adsorbate species penetrated deeper into the Ac-NWHHC through the wider inter-granular pores created upon activation.^93,94^

#### 3.2.5 Adsorption kinetics of CV dye and Cr(vi)

**Fig. 6:**
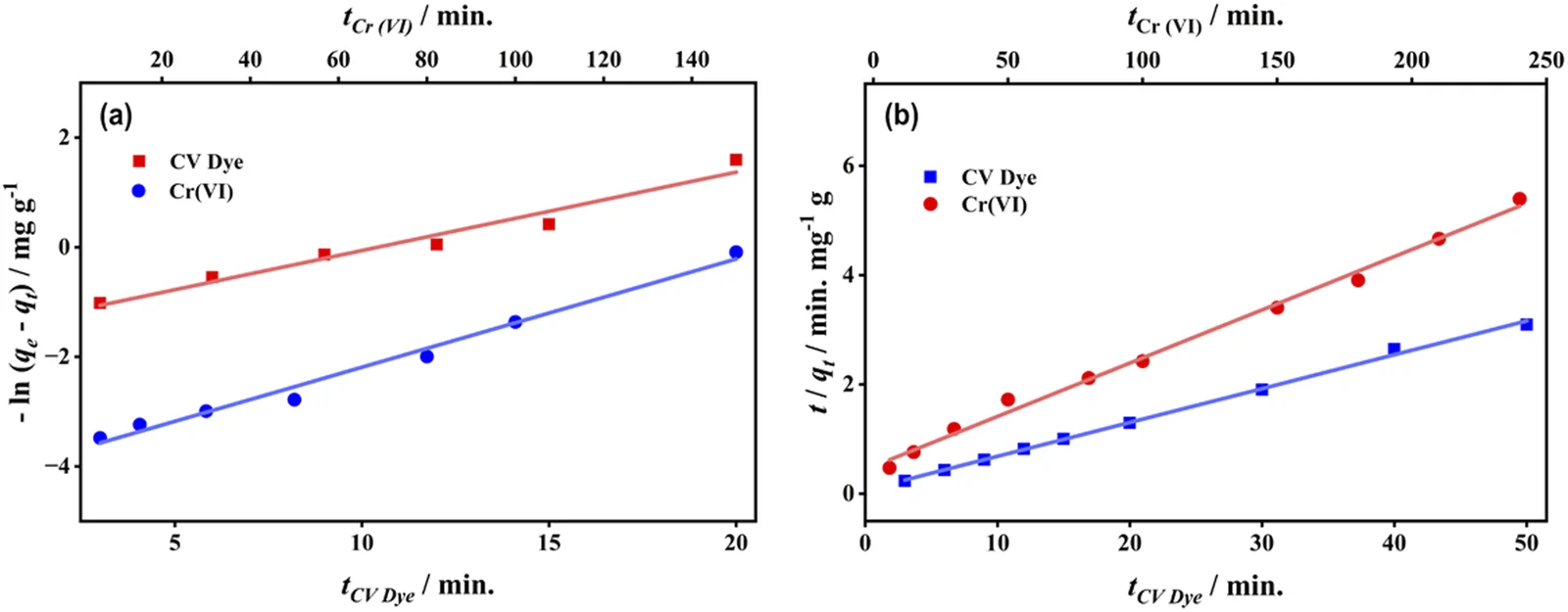
The linear plots of the (a) pseudo-first-order (PFO) and (b) pseudo-second-order (PSO) models for the adsorption of CV dye and Cr(vi) onto Ac-NWHHC.

The linear plots derived from the kinetic equations are presented in Fig. 6, along with the kinetic parameters compiled in Table 1. For the sorption of both adsorbates (CV dye and Cr(vi)), the pseudo-second-order (PSO) kinetic model (Fig. 6b) yielded a higher adjusted determination coefficient, *R*_adj._^2^ Compared to the Pseudo-first-order (PFO) kinetic model (Fig. 6a). Moreover, the PSO kinetic equation predicted the *q*_e(theoretical)_ of both the CV dye and Cr(vi) onto Ac-NWHHC closer to the *q*_e(experimental)_, obtained from the experimental investigations. Hence, the PSO kinetic model can be considered as the best fit to describe the kinetics associated with the adsorption of CV dye and Cr(vi) onto Ac-NWHHC. Hence, it can be assumed that the rates of the corresponding adsorption processes were primarily governed by chemical interactions, such as the n–π or π–π type interactions of the dye molecules with the doped N atoms and the oxygenous functional groups, the chemical reduction of Cr(vi) to Cr(iii) by the surface functionalities, *etc.* Physical interactions, such as electrostatic attractions, hydrogen bonding, *etc.*, could also have contributions to the overall rate of adsorption, given the acceptable *R*_adj._^2^ value obtained for the PFO kinetic model.^54,95^ Here, the appreciable *R*_adj._^2^ of the PFO kinetic model in the case of both adsorbates could be attributed to the initial stage of adsorption, where the rapid mass transfer of the adsorbate species took place as the PFO model is best fitted to describe the initial stage of an adsorption process when the adsorbate concentration is high.^45^

**Table 1:** The kinetic parameters obtained for the adsorption of CV dye and Cr(vi) onto Ac-NWHHC. The solution conditions were pH = 6.3 (CV dye) and 2.0 (Cr(vi)), *C*_o_ = 10 mg L^−1^ (CV dye) and 30 mg L^−1^ (Cr(vi)), adsorbent dose = 0.5 g L^−1^, and temperature = room temperature

Kinetic models	Parameters	CV dye	Cr(vi)
PFO	*R*_adj._^2^	0.95	0.98
	*k*_1_ (min^−1^)	0.14	0.02
	*q*_e_ (cal.) (mg g^−1^)	4.44	40.95
	*q*_e_ (exp.) (mg g^−1^)	16.16	46.07
PSO	*R*_adj._^2^	0.99	0.99
	*k*_2_ (g mg^−1^ min^−1^)	0.06	7.87 × 10^−4^
	*q*_e_ (cal.) (mg g^−1^)	16.14	50.40
	*q*_e_ (exp.) (mg g^−1^)	16.16	46.07
Elovich	*R*_adj._^2^	0.98	0.96
	*a* (mg g^−1^ min^−1^)	2.36 × 10^4^	5.72
	*b* (g mg^−1^)	0.84	0.11
W–M	*R*_adj._^2^	0.985	0.99
	*k*_id_ (mg g^−1^ min^−0.5^)	1.29	3.68

The Elovich kinetic equation (Fig. S5a) also showed a high *R*_adj._^2^ for the adsorption of both adsorbate species, indicating the heterogeneity of the Ac-NWHHC surface.^96^ The higher value of *a* yielded by the Elovich kinetic model for the CV dye sorption provides further evidence, supporting earlier observation of rapid dye uptake at the initial stage in Fig. 4c. But the value of *a* for Cr(vi) was lower, indicating the brisk but steady uptake of the Cr(vi) species at the beginning of the adsorption process, which is consistent with the trend in Cr(vi) removal at different time intervals observed in Fig. 4d. Fitting of both the adsorbates' adsorption data to the Weber–Morris kinetic equation in Fig. S5b was resolved into three stages, indicating that intra-particle diffusion is not the sole rate-limiting step.^97^ The initial steep slope of the first stage indicates the rapid external mass transfer of the adsorbates from the bulk solution onto the outer surface of Ac-NWHHC, driven by the strong concentration gradient. This was followed by the gradual diffusion of the adsorbates through the macropores, during which the adsorbates diffused into the porous channel of Ac-NWHHC and interacted with the active sites. Later, the adsorbates penetrated further inside the adsorbent, approaching adsorption equilibrium.^98,99^

#### 3.2.6 Adsorption isotherms of CV dye and Cr(vi) onto Ac-NWHHC

**Fig. 7:**
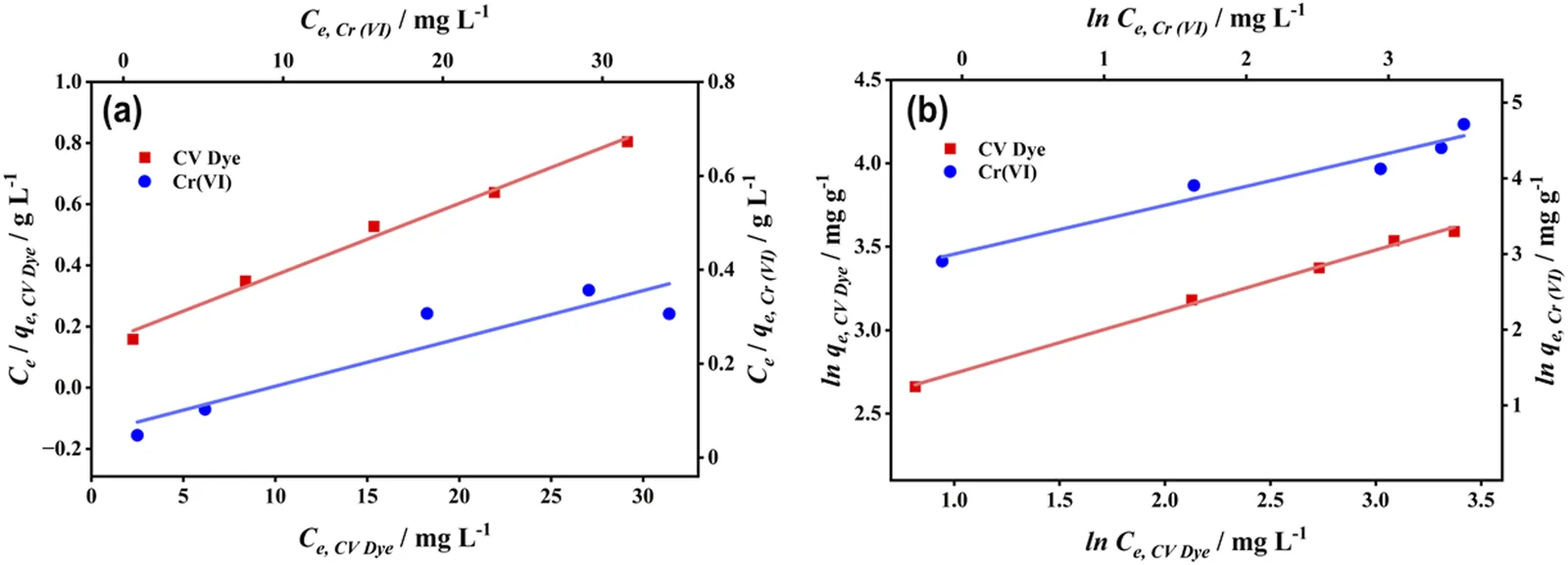
The linear fitting of the (a) Langmuir and (b) Freundlich isotherm models for the adsorption of CV dye and Cr(vi) onto Ac-NWHHC.

Fig. 7a and b presents the linear regression plots of the Langmuir and Freundlich adsorption isotherm models, and Fig. S6 presents the Temkin isotherm plot for CV dye and Cr(vi) onto Ac-NWHHC. All the parameters derived from the isotherm equations are summarized in Table 2. Higher *R*_adj._^2^ values were obtained for the Freundlich isotherm model for both adsorbates, indicating the heterogeneity of the Ac-NWHHC surface, as well as the accumulation of the adsorbates onto the Ac-NWHHC surface *via* forming successive layers. Earlier, the structural characterization revealed a diverse array of O and N-functional groups on the surface of Ac-NWHHC, which possess different binding energies due to their different chemical environments and properties. The Freundlich isotherm parameters obtained for both adsorbates also predicted the favorability of the adsorption onto Ac-NWHHC. For both adsorbates, a larger *K*_F_ value was observed, indicating a higher adsorption capacity of Ac-NWHHC under the studied conditions.^100^ The adsorption intensity constant, *n*, is also indicative of adsorption favorability and surface heterogeneity. A value of 1/*n* < 0.5 suggests a high degree of preference for an adsorption process. For both adsorbates, the values of 1/*n* < 0.5 suggest strong adsorbate–adsorbent interactions.^33^ From the Langmuir isotherm study, a maximum monolayer adsorption capacity (*q*_max_) of ∼42.63 and ∼113.12 mg g^−1^ were obtained for CV dye and Cr(vi) onto Ac-NWHHC, respectively. Since the Langmuir adsorption isotherm also gave an appreciable *R*_adj._^2^ particularly for the CV dye sorption, it can be assumed that CV dye molecules formed a certain extent of monolayers onto the Ac-NWHHC surface.^101^ The values of the Temkin constant (*B*_T_) for CV dye and Cr(vi) adsorption were obtained as 8.66 and 20.85 kJ mol^−1^, respectively, which are associated with the heat of adsorption. This suggested that both the CV dye and Cr(vi) adsorption were predominantly driven by physical interactions.^102^

**Table 2:** The adsorption isotherm parameters obtained for the adsorption of CV dye and Cr(vi) onto Ac-NWHHC. The solution conditions were pH = 6.3 (CV dye) and 2.0 (Cr(vi)), *C*_o_ = 10–50 mg L^−1^ (CV dye) and 10–90 mg L^−1^ (Cr(vi)), adsorbent dose = 0.5 g L^−1^, and temperature = room temperature

Isotherm models	Parameters	CV dye	Cr(vi)
Langmuir	*R*_adj._^2^	0.986	0.86
	*K*_L_ (L mg^−1^)	0.18	0.13
	*q*_max._ (mg g^−1^)	42.63	113.12
Freundlich	*R*_adj._^2^	0.995	0.95
	*K*_F_ (L mg^−1^)	10.71	20.54
	1/*n*	0.37	0.44
Temkin	*R*_adj._^2^	0.984	0.84
	*K*_T_ (L mg^−1^)	2.15	2.30
	*B*_T_ (KJ mol^−1^)	8.66	20.85

To evaluate the effectiveness of Ac-NWHHC for the removal of CV dye and Cr(vi), its adsorption performance was compared with some previously reported bioadsorbents and is summarized in Table 3. Comparing the adsorption capacities of the selected adsorbents with that of Ac-NWHHC, it can be suggested that the proposed two-stage hydrothermal synthetic route used in this work successfully prepared the N-doped activated hydrochar which displayed an appreciable adsorption performance for both the CV dye and Cr(vi). The employment of the two separate hydrothermal treatment strategies in this work not only preserved the various functional states of the incorporated N-atoms and the biomass-derived oxygen functionalities but also led to the formation of a mesoporous quasi-spherical structure of the adsorbent, which combinedly gave an enhanced removal capacity of Ac-NWHHC.

**Table 3:** Comparative adsorption capacities of Ac-NWHHC with previously reported bio-adsorbents for CV dye and Cr(vi)

Adsorbate	Adsorbent	Adsorbent dose, g L^−1^	Maximum adsorption capacity, mg g^−1^	References
CV dye	Palm kernel shell-derived biochar	16.67	24.45	85
	Lemon wood-derived activated carbon-Fe_3_O_4_ nanocomposite	1.25	35.31	103
	N self-doped hydrochar from Siam weed	2	37.47	104
	Pine cone biochar modified with CoFe_2_O_4_/Mn–Fe LDH	1	41.152	102
	Activated carbon from food waste *via* ZnCl_2_ activation	1.18	57.9	105
	ZnCl_2_-activated N-doped water hyacinth hydrochar	0.5	42.63	This study
Cr(vi)	Mesoporous hydrochar from *Acacia falcata* leaves	0.4	30.47	106
	ZnCl_2_-modified bamboo sawdust hydrochar	3.3	14.0	107
	H_2_SO_4_-treated poultry litter-derived hydrochar	2	26.21	108
	Fe-rich wheat straw hydrochar	1.25	116.29	109
	Fe and N-doped pinewood sawdust biochar	1	127.8	110
	ZnCl_2_-activated N-doped water hyacinth hydrochar	0.5	113.12	This study

#### 3.2.7 Adsorption thermodynamics of CV dye and Cr(vi) onto Ac-NWHHC

**Fig. 8:**
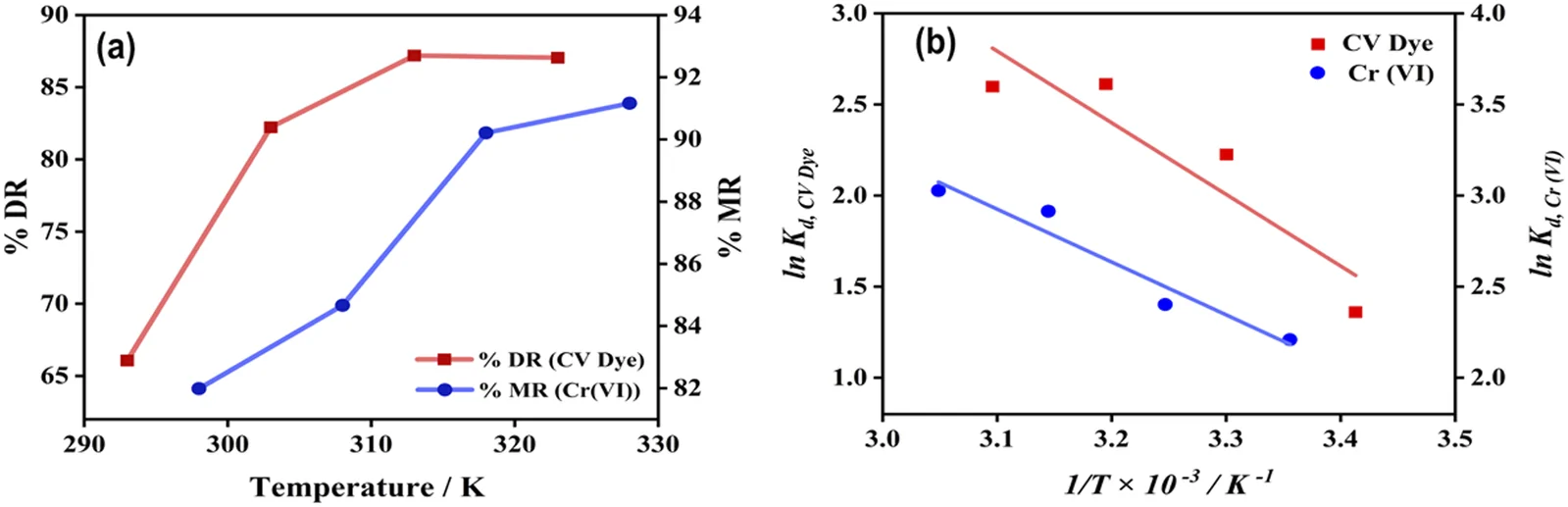
(a) Effect of temperature on the CV dye and Cr(vi) removal efficiency of Ac-NWHHC, and (b) The van't Hoff Plot for the adsorption of CV dye and Cr(vi) at different temperatures.

Fig. 8a represents the thermodynamic investigation of the % of CV dye and Cr(vi) uptake onto Ac-NWHHC adsorbent at different temperatures. The thermodynamic parameters for CV dye and Cr(vi) adsorption processes were evaluated from the van't Hoff plot [Fig. 8b] and are summarized in Table 4. A significant increase in the % of removal of both adsorbates was observed as the temperature increased [Fig. 8a]. This positive temperature dependence suggests that higher temperatures promoted the adsorption of CV dye and Cr(vi) onto Ac-NWHHC and hence both can be regarded as endothermic processes.^111,112^ The enhanced adsorption at higher temperatures can be attributed to several factors: at elevated temperatures, the mobility of the adsorbate species increased. This gained kinetic energy not only promoted the frequency of collisions with the surface active sites on Ac-NWHHC but also favored the diffusion of the CV dye molecules and Cr(vi) species into the porous structure of Ac-NWHHC, thereby increasing the overall adsorption capacity.^113,114^ Indeed, the supplied thermal energy helped overcome the energy barrier associated with adsorption. The negative values of the Gibbs free energy changes (Δ*G*°) indicated that the adsorptions of CV dye and Cr(vi) onto Ac-NWHHC were thermodynamically favorable.^33,115^ The standard enthalpy (Δ*H*°) and entropy (Δ*S*°) changes for both adsorbates were derived from the intercept and the slope of the van't Hoff plot [Fig. 8b], respectively, where the positive enthalpy changes (Δ*H*° > 0) for both adsorbates further support the endothermic nature of both adsorption processes.^116^ The Δ*H*° values for the CV dye and Cr(vi) adsorption onto Ac-NWHHC are 32.71 and 24.14 kJ mol^−1^, respectively. The large positive entropy changes (Δ*S*° > 0), along with the negative Δ*G*°, indicate that the adsorption processes were predominantly entropy-driven, reflecting the increased disorder at the adsorbent/solution interface during adsorption as temperature was increased.^92^

**Table 4:** The thermodynamic parameters obtained for the adsorption of CV dye and Cr(vi) onto Ac-NWHHC at different temperatures. The solution conditions were pH = 6.3 (CV dye) and 2.0 (Cr(vi)), *C*_o_ = 10 mg L^−1^ (CV dye) and 30 mg L^−1^ (Cr(vi)), and adsorbent dose = 0.5 g L^−1^

Adsorbates	Temperature (K)	Δ*G*° = −*RT* ln *K*_d_ (kJ mol^−1^)	Δ*H*° (kJ mol^−1^)	Δ*S*° (J mol K^−1^)
CV dye	293	−3.31	32.71	124.63
	303	−5.61		
	313	−6.80		
	323	−6.98		
Cr(vi)	298	−5.47	24.14	99.16
	308	−6.15		
	318	−7.71		
	328	−8.26		

#### 3.2.8 Effect of coexisting ions on CV dye and Cr(vi) removal

**Fig. 9:**
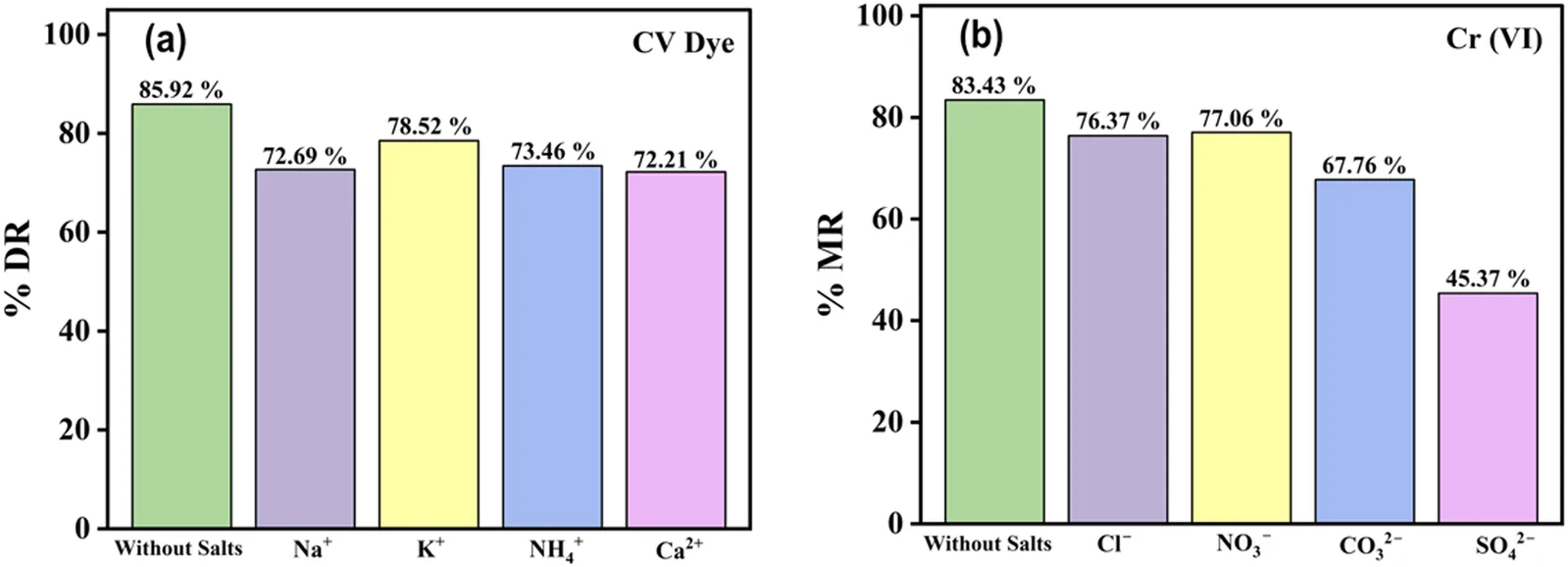
Effect of coexisting (a) cations on the adsorption of CV dye and (b) anions on the adsorption of Cr(vi) onto Ac-NWHHC, where the coexisting ions' concentration = 0.1 M, pH = 6.3 (CV dye) and 2.0 (Cr(vi)), *C*_o_ = 10 mg L^−1^ (CV dye) and 30 mg L^−1^ (Cr(vi)), and adsorbent dose = 0.5 g L^−1^.

To verify the adsorption selectivity of Ac-NWHHC, its sorption efficiency for both adsorbates was investigated in the presence of several coexisting ions in the aqueous system as industrial wastewater contains a variety of inorganic contaminants. For CV dye, four different cations (Na^+^, K^+^, NH_4_^+^, and Ca^2+^) were dissolved separately in the solution, maintaining a concentration of 0.1 M for each ion. From Fig. 9a, it can be observed that the dye removal efficiency of Ac-NWHHC decreased due to the presence of the cations. The observation could be attributed to the competitive sorption of the hydrated cations on the adsorbent surface, which could block the binding sites on Ac-NWHHC.^85^ The effect of the dissolved cations on the removal of CV dye onto Ac-NWHHC exhibited the following trend: Ca^2+^ > Na^+^ > NH_4_^+^ > K^+^. The divalent Ca^2+^ ions exhibit stronger electrostatic interactions with the negatively charged active sites, resulting in stronger competition with the cationic CV dye molecules. However, Ac-NWHHC removed more than 70% of the CV dye from the solution, competing with the coexisting cations.

For Cr(vi), four different anions (Cl^−^, NO_3_^−^, CO_3_^2−^, and SO_4_^2−^) of 0.1 M concentration were added to the metal ion solution to assess the selectivity of Ac-NWHHC for the negative HCrO_4_^−^ species at acidic pH. From Fig. 9b, a decreasing trend in the removal efficiency of Ac-NWHHC was observed for Cr(vi) as well. As the negative charge of the coexisting anion increased, the removal efficiency decreased accordingly. Again, this reduction in the Cr(vi) removal could be attributed to the competition of the dissolved anions for the active sites on Ac-NWHHC, which affected the sorption of the negatively charged Cr(vi) species.^109^ Here, the Cl^−^ and NO_3_^−^ had minimal effect, but the CO_3_^2−^ and SO_4_^2−^ ions significantly decreased the removal, as those possess a negative charge compared to the negatively charged Cr(vi) species (CrO_4_^−^), making them strong competitors for the active sites. Even though the coexisting ions influenced the adsorption performance of Ac-NWHHC to varying degrees due to strong competition of the ions for the adsorption sites, Ac-NWHHC maintained an appreciable removal efficiency for the target pollutants (CV dye and Cr(vi)), demonstrating its feasibility in real wastewater treatment.

#### 3.2.9 Reusability of Ac-NWHHC

**Fig. 10:**
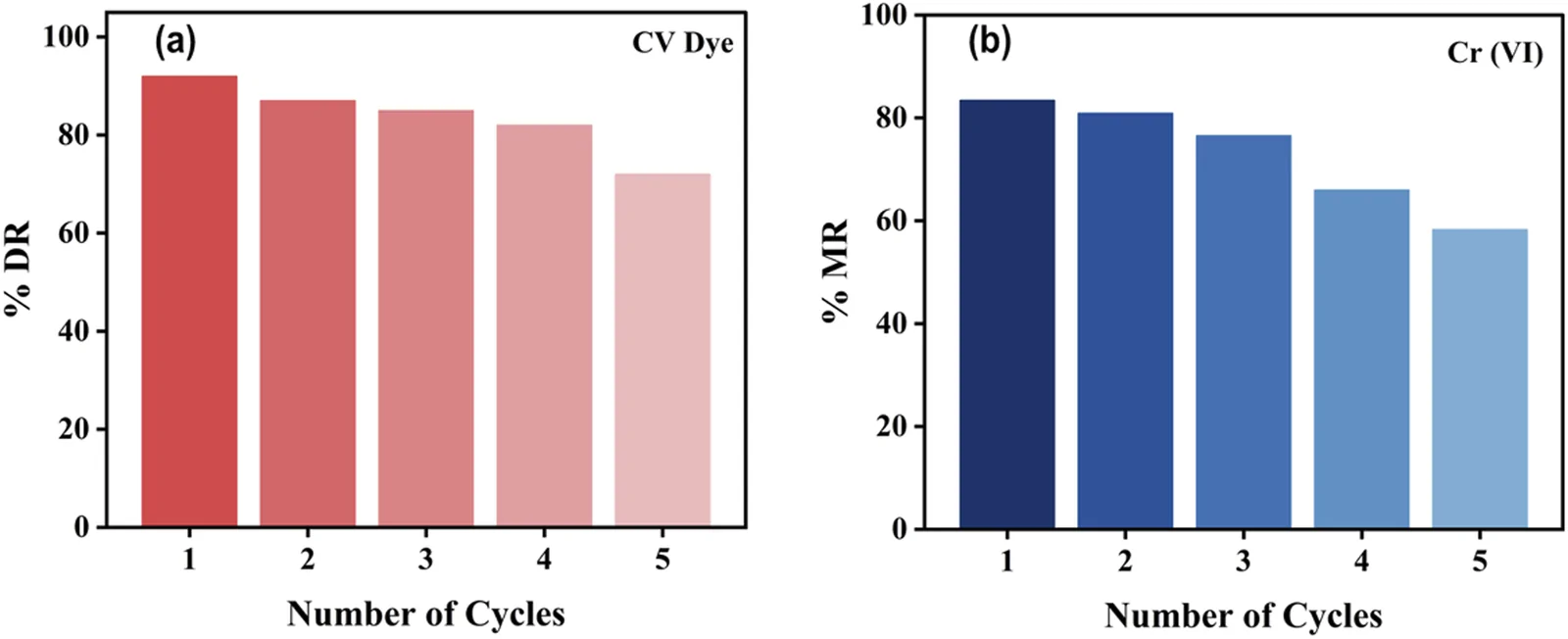
Reusability of Ac-NWHHC for (a) CV dye and (b) Cr(vi) for five cycles. Here *C*_o_ = 10 mg L^−1^ (CV dye) and 30 mg L^−1^ (Cr(vi)), and adsorbent dose = 0.5 g L^−1^, volume = 10 mL.

The reusability of Ac-NWHHC was investigated [Fig. 10(a and b)] for both adsorbates to assess its stability, regeneration efficiency, long-term performance, as well as economic feasibility. Here, the adsorbent was utilized for five consecutive cycles for individual removal of CV dye and Cr(vi). The pH-based desorption method was used to regenerate the adsorbate loaded Ac-NWHHC. For each cycle, the adsorption of CV dye was performed at solution pH, 6.3 and desorption was conducted by altering the solution pH to acidic pH, 2.0 by using 0.1 M HCl at room temperature. A similar procedure was employed for checking the reusability of Ac-NWHHC for removing Cr(vi), but desorption of Cr(vi) was conducted in basic solution of pH approximately 8.0, as its maximum adsorption occurred at pH 2.0. After each cycle, the adsorbent was washed thoroughly with deionized water until neutral pH was achieved, collected by centrifugation and oven dried prior to be reused for subsequent cycles. Ac-NWHHC removed almost 92% of CV dye and 83.4% of Cr(vi) in the first cycle. After reusing it for five cycles, the removal % of CV dye and Cr(vi) were 72% and 58.29%, respectively. For both adsorbates, Ac-NWHHC retained more than 50% of its initial sorption efficiency with a minimal adsorbent dosage of 0.5 g L^−1^. The gradual decrease in the removal efficiency of Ac-NWHHC could be attributed to incomplete recovery of the functional groups or the active sites during the desorption process for multiple cycles.^104,106^ As the adsorbent was reutilized for several cycles, there could be bonding sites blocked by the adsorbed adsorbates from the previous cycles, which led to the reduction of the removal efficiency. Yet, Ac-NWHHC exhibited appreciable sorption capability even after five consecutive cycles, highlighting its structural stability and suitability for practical wastewater treatment.

### 3.3 Post-adsorption characterization of Ac-NWHHC for adsorption mechanism

**Fig. 11:**
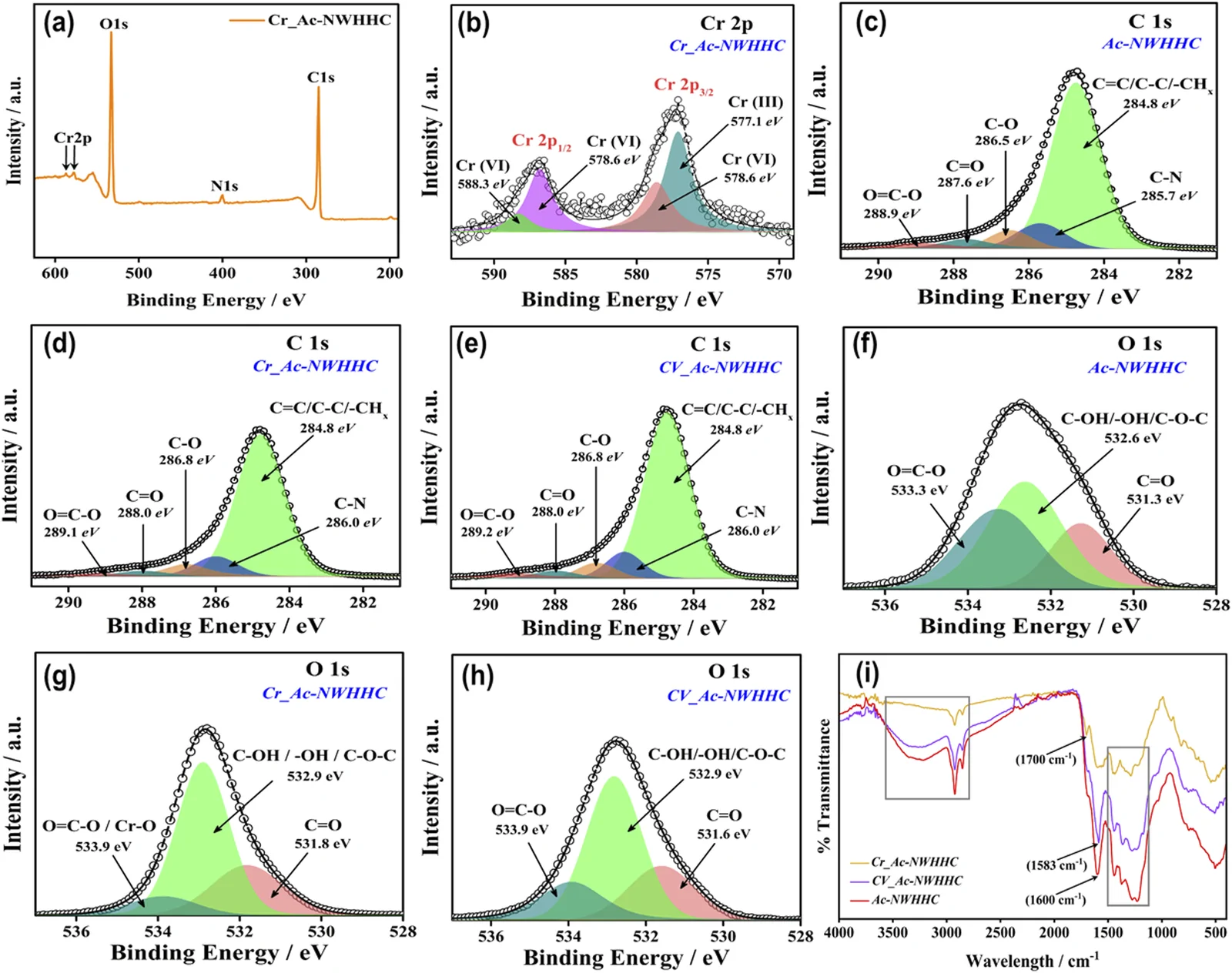
(a) The XPS survey of Ac-NWHHC after Cr(vi) adsorption, (b) the deconvoluted Cr 2p XPS spectra of Cr_Ac-NWHHC. The deconvoluted C 1s (c–e) and the O 1s XPS spectra (f–h) of Ac-NWHHC, Cr_Ac-NWHHC, and CV_Ac-NWHHC, respectively. (i) The FTIR spectra of Ac-NWHHC before and after adsorption.

To gain insight into the adsorption mechanism of CV and Cr(vi) onto the Ac-NWHHC surface, XPS and FTIR spectra were examined before and after the adsorption process. Fig. 11a and b represent the wide-scan XPS survey and deconvoluted Cr 2p fine-scale spectrum of the Ac-NWHHC after adsorbing Cr(vi) ions (Cr_Ac-NWHHC), respectively. The presence of the Cr 2p doublet peak in the XPS survey of Ac-NWHHC after Cr(vi) ion adsorption confirms the successful adsorption of Cr(vi) onto the Ac-NWHHC surface. The deconvoluted Cr 2p fine-scale spectrum [Fig. 11b] showed four major peaks in the binding energies of 577.1 and 586.8 eV for Cr(iii) and at 578.6 and 588.3 eV for Cr(vi) species.^117^ The presence of Cr(iii) species in the Cr 2p spectrum might be due to the surface reduction of Cr(vi) to Cr(iii) during adsorption, indicating the adsorption-reduction mechanism of Cr(vi). The reduction of Cr(vi) species might occur according to the following equation.^118^

10 HCrO_4_^−^ (aq.) + 7H^+^ (aq.) + 3e^−^ → Cr^3+^ (aq.) + 4H_2_O (l)

The deconvoluted C 1s XPS spectra of Ac-NWHHC before Cr(vi) adsorption showed five peaks for C=C/C–C/–CH, C–N, C–O, C=O, and O–C=O groups at around 284.8, 285.7, 286.5, 287.6, and 288.9 eV, respectively [Fig. 11c]. It was observed that the binding energy for C=C/C–C/–CH remained unchanged after the adsorption of Cr(vi) onto the Ac-NWHHC surface, whereas C–N, C–O, C=O, and O–C=O groups showed a positive binding energy shift (Fig. 11d). A similar response of the C 1s XPS spectrum was also observed after the adsorption of CV dye molecules onto the Ac-NWHHC surface (CV_Ac-NWHHC), as shown in Fig. 11e, reflecting the involvement of C–N, C–O, C=O, and O–C=O functional groups in the adsorption of both adsorbate species. The O 1s XPS spectra provide vital information on the involvement of oxygen functionalities in the adsorption process. The Ac-NWHHC sample exhibits three major peaks at around 531.3, 532.6 and 533.3 eV corresponding to the carbonyl oxygen (C=O), hydroxyl or ether groups and carboxylic ester, respectively (Fig. 11f). Following both Cr(vi) and CV dye adsorption, noticeable peak shifts were observed for C=O (∼531.8 eV), –OH or O–C–O (532.9 eV) and O=C–O (533.9 eV) species as shown in Fig. 11 (g and h), indicating that these oxygen containing groups might have electrostatic interaction with CV dye and Cr ions at their respective adsorption experimental conditions. In addition, the higher binding energy peak at 533.9 eV suggests electron donation and metal–oxygen coordination bond formation possibilities of oxygen functional groups during the Cr(vi) adsorption process.^119^ Similar changes were also observed in the N 1s spectra of CV_Ac-NWHHC and Cr_Ac-NWHHC, as shown in Fig. S7. The N 1s XPS spectra of Ac-NWHHC exhibit three major peaks for pyridinic, pyrrolic and graphitic-N at around 398.9, 400.1 and 401.1 eV, respectively. In contrast, the N 1s spectra of CV_Ac-NWHHC and Cr_Ac-NWHHC display four peaks at comparatively higher binding energies except for graphitic-N. These positive binding energy shifts for pyrrolic and pyridinic-N moieties indicate that both the pyrrolic and pyridinic-N moieties can act as Lewis bases (donating electrons) with Cr(vi) and CV dye molecules. A new peak was observed at 401.9 eV after the adsorption of Cr(vi) and CV dye, suggesting the formation of protonated or quaternary-N species and electron donation to the Cr(vi) ion to form a metal–N complex.^117,119^ On the other hand, C–O and C–N groups are expected to donate electrons and significantly reduce the Cr(vi) species. The π electrons in the C=C moieties of the Ac-NWHHC matrix are also expected to actively facilitate the Cr(vi)/Cr(iii) reduction.^120^

11 C-ÖH/C–N/C=C + Cr(vi) → Cr(iii) [Reduction]

Moreover, the surface positive charge at the acidic pH (∼pH 2) induced strong electrostatic interactions between the Cr(vi) anions and the protonated functional groups.

The FTIR spectra of Ac-NWHHC also exhibited significant shifts and changes after CV dye and Cr(vi) adsorption [Fig. 11i]. The aromatic C=C stretch at ∼1600 cm^−1^ shifted to a lower wavenumber of 1583 cm^−1^ in CV_Ac-NWHHC. A significant decrease in the intensity of the C=C band was observed in Cr_Ac-NWHHC. The π electrons in the aromatic fragments of the Ac-NWHHC could lead to π–π type interactions with the aromatic system in the CV dye molecules, and could also reduce the Cr(vi) ions.^87,116^ The broad overlapped peaks observed in the range of 1230–1276 cm^−1^ for the heterocyclic-N also exhibited significant changes after adsorption, which was evident from the XPS analyses [Fig. 11a–e]. The sharpening of the C=O stretching band in Cr_Ac-NWHHC at 1700 cm^−1^ can be attributed to the oxidation of the -C-O^−^ upon reducing the adsorbed Cr(vi) to Cr(iii).^121^ The weakened intensity of the –OH stretching peak indicated the adsorbate molecules' interaction with the surface –OH functional groups. The C–O–H groups can form H-bonds with the dye molecules, as well as inner–sphere complexes with the Cr(vi).^122^

**Fig. 12:**
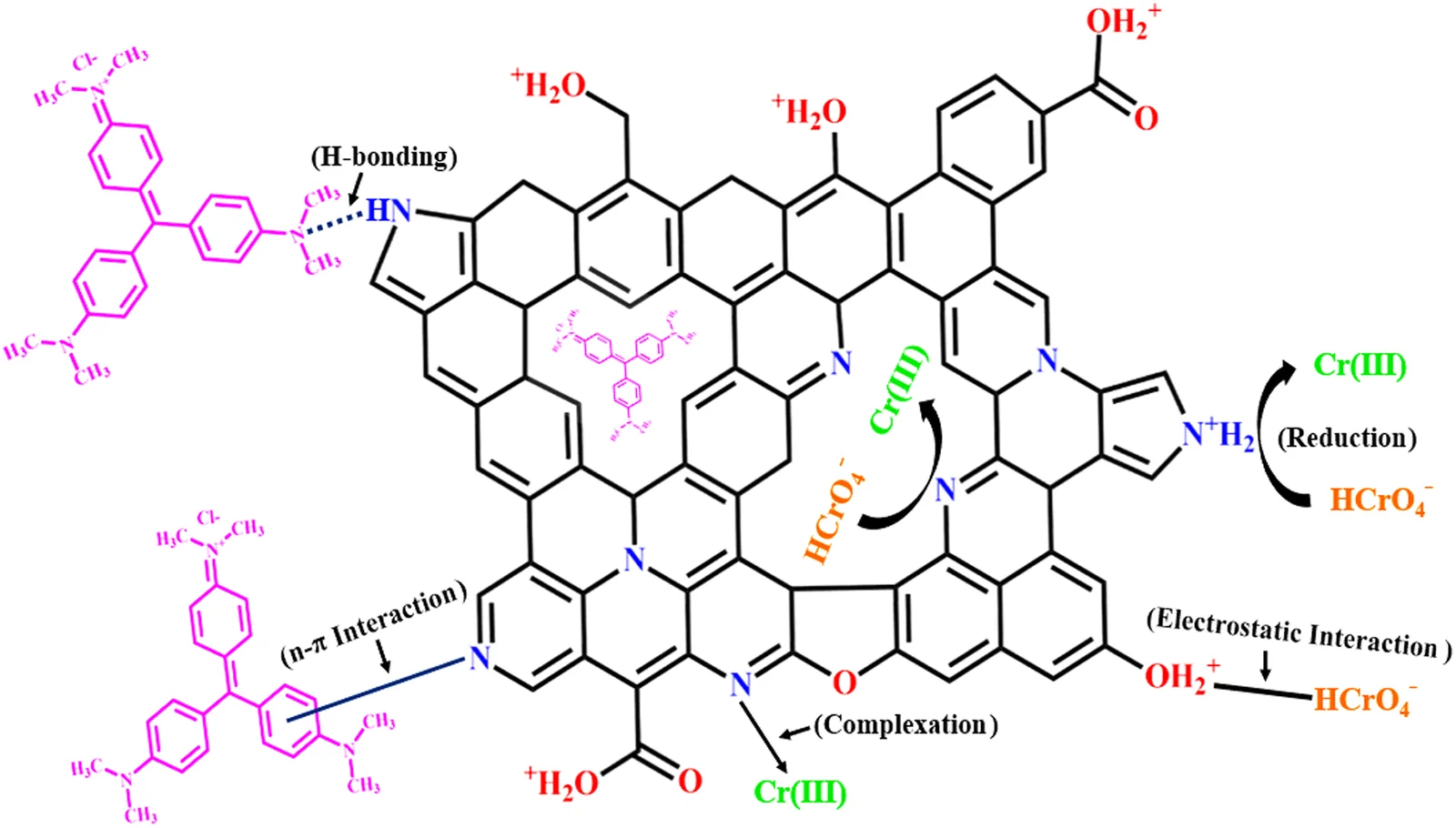
Visual illustration of the proposed different adsorption mechanisms of the adsorbates onto Ac-NWHHC.

A visual representation of the proposed possible adsorption mechanisms of the corresponding adsorbates onto Ac-NWHHC is shown in Fig. 12.

## 4. Conclusion

The N-incorporated activated hydrochar from biomass using a two-stage hydrothermal strategy was successfully synthesized and resulted in an appreciable retention of the doped N even after the activation process. The adsorbent was characterized by XPS, FTIR, FESEM, EDS, BET, and TGA methods. Successful incorporation of N atoms is confirmed by XPS analyses, where N atoms were found to be present in the pyrrolic, pyridinic, and graphitic formations in the Ac-NWHHC matrix. The BET isotherm confirmed that the surface area increased by approximately 7.7 times after the chemical activation of NWHHC. Additionally, the FESEM analysis further verified the formation of quasi-spherical granular morphology of the Ac-NWHHC from the sheet-like surface of NWHHC. The Ac-NWHHC was found to adsorb ∼85.4% of crystal violet dye and ∼81.2% of Cr(vi) from their corresponding aqueous solutions. The kinetic studies revealed that both adsorbates' adsorption processes were predominantly followed by the PSO model, confirming the involvement of various physical and chemical adsorbate–adsorbent interactions. The Freundlich isotherm was found to best fit the experimental data of both adsorbates, indicating the dominance of multilayer adsorption process on the heterogeneous surface. The post-adsorption characterization using XPS and FTIR further supported these findings. The shifting of the binding energies and reduction of the absolute areas in the XPS spectra of the adsorbent after adsorption revealed the involvement of various surface functional groups in the uptake of both adsorbates, where hydrogen bonding, π–π interactions, and n–π interactions were dominant. The Langmuir isotherm provided the maximum monolayer adsorption capacities of Ac-NWHHC to be 42.63 mg g^−1^ for CV dye and 113.12 mg g^−1^ for Cr(vi) removal. The thermodynamic analysis showed that the adsorption process is thermodynamically favorable (Δ*G* < 0) and endothermic in nature (Δ*H* > 0) for both adsorbates. Further, the reusability test demonstrated efficient adsorption capacity over several regeneration cycles, suggesting the functional and structural integrity preferable for practical applications. The results obtained from this work demonstrated that the synthesized Ac-NWHHC from naturally abundant water hyacinth possesses significant potential for wastewater remediation. The low-cost precursor, simple synthesis, and chemical modification make Ac-NWHHC an efficient, environmentally friendly, and sustainable adsorbent, holding considerable promise for large-scale wastewater treatment.

## Author contributions

Md. Hassan Shahriar: methodology, data curation, formal analysis, investigation, visualization, writing – original draft. Nahida Akter: conceptualization, supervision, formal analysis, validation, writing – review & editing. Md. Tanvir Shahriar: data curation, formal analysis, investigation. Tasfia Jaman Ahana: data curation, investigation. Md. Mahadi Hasan: data curation, investigation. Md. Rezwan Miah: validation, writing – review & editing. S. M. Nizam Uddin: conceptualization, formal analysis, funding acquisition, supervision, validation, visualization, writing – review & editing.

## Conflicts of interest

The authors declare that they have no known competing financial interests or personal relationships that could have appeared to influence the work reported in this paper.

## Supplementary Material

RA-OLF-D6RA07071J-s001

## Data Availability

The data related to this work are available in the article and in the supplementary information (SI) file. Additional data related to this study are available upon reasonable request from the corresponding authors. Supplementary information: high-resolution O 1s XPS spectra and EDX analysis of NWHHC, and Ac-NWHHC, N_2_ adsorption–desorption isotherm of NWHHC, determination of the point of zero charge of Ac-NWHHC, linear plots of the Elovich, W–M kinetic and Temkin adsorption isotherm models for the adsorption of CV dye and Cr(vi) onto Ac-NWHHC, high-resolution N 1s XPS spectra of Ac-NWHHC, CV_Ac-NWHHC, and Cr_Ac-NWHHC. See DOI: https://doi.org/10.1039/d6ra07071j.
