# Supplementary material for: Surface engineering of ZnCl2-activated N-doped water hyacinth hydrochar: a mechanistic insight for fast and efficient removal of Cr(vi) and crystal violet dye
Source: RSC Adv. 2026 Sep 8;16(43):53323–40. doi: 10.1039/d6ra07071j (PMC13551272; doi:10.1039/d6ra07071j)
Supplement: Supplementary file 1 [file RA-016-D6RA07071J-s001.pdf]

Supplementary Information (SI) for RSC Advances.
This journal is © The Royal Society of Chemistry 2026

Supporting Information

Surface Engineering of $ZnCl_2$-Activated N-Doped Water Hyacinth Hydrochar: A Mechanistic Insight for Fast and Efficient Removal of Cr (VI) and Crystal Violet Dye

Md. Hassan Shahriar[a], Nahida Akter[b]*, Md. Tanvir Shahriar[a], Tasfia Jaman Ahana[a], Md. Mahadi Hasan[a], Md. Rezwan Miah[a], S. M. Nizam Uddin[a]*

[a]*Department of Chemistry, Graduate School of Physical Sciences, Shahjalal University of Science and Technology, Sylhet-3114, Bangladesh*

[b]*Department of Chemistry, University of Barishal, Barishal-8254, Bangladesh*

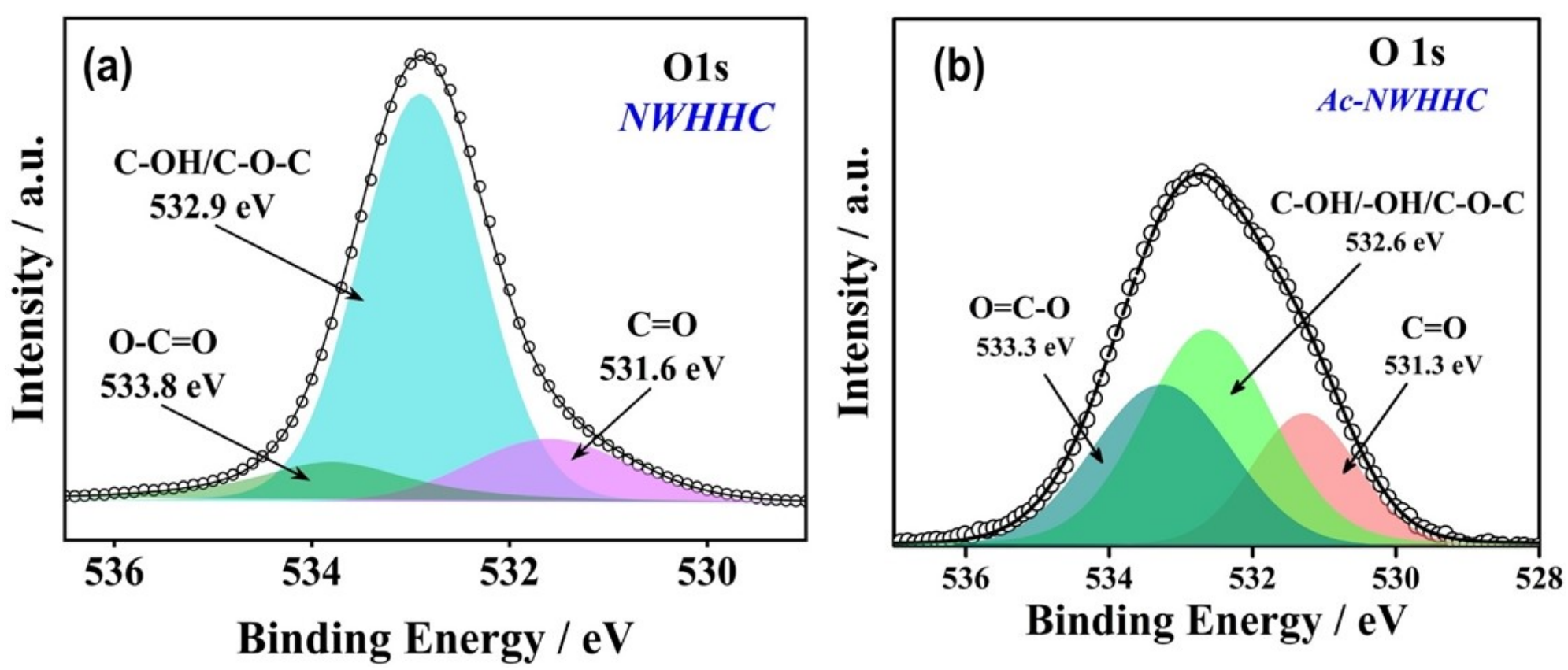

Fig. S1. High-resolution O 1s XPS spectra of (a) NWHHC, and (b) Ac-NWHHC.

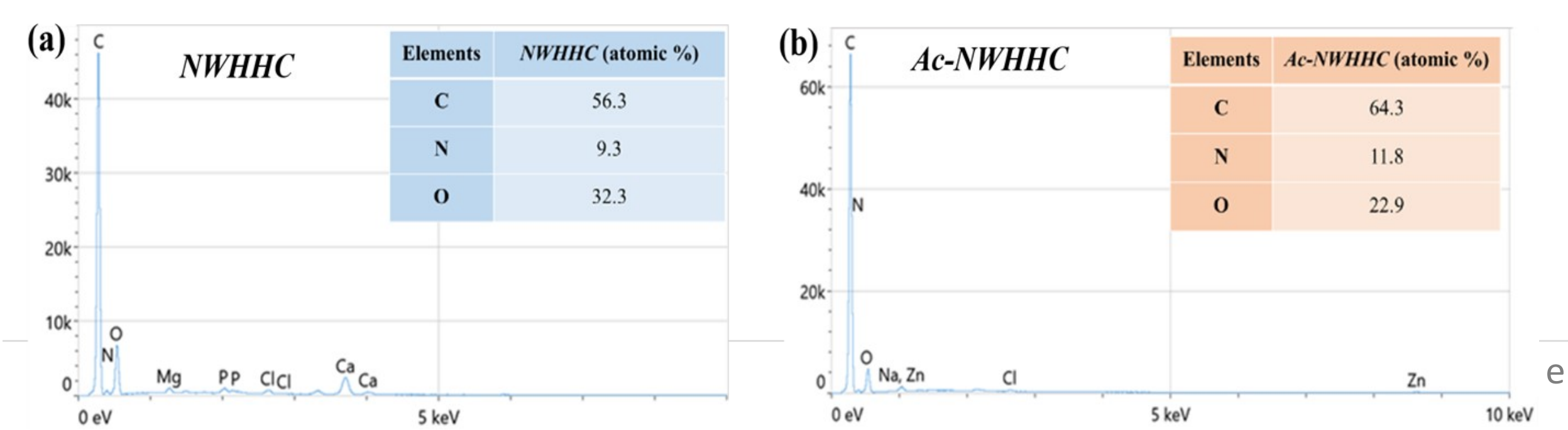

Fig. S2. EDS analysis of (a) NWHHC, and (b) Ac-NWHHC.

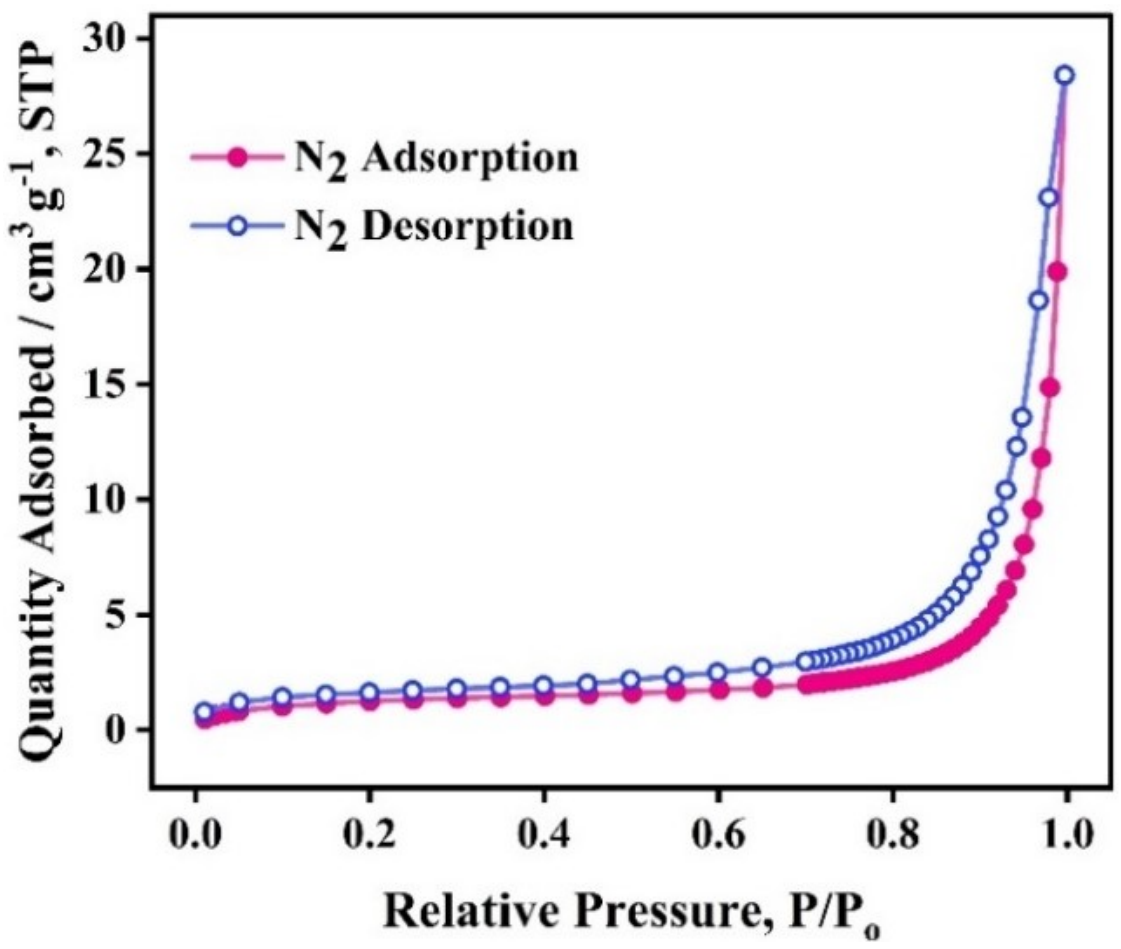

Fig. S3. N_2 adsorption-desorption isotherm of NWHHC at 77 K.

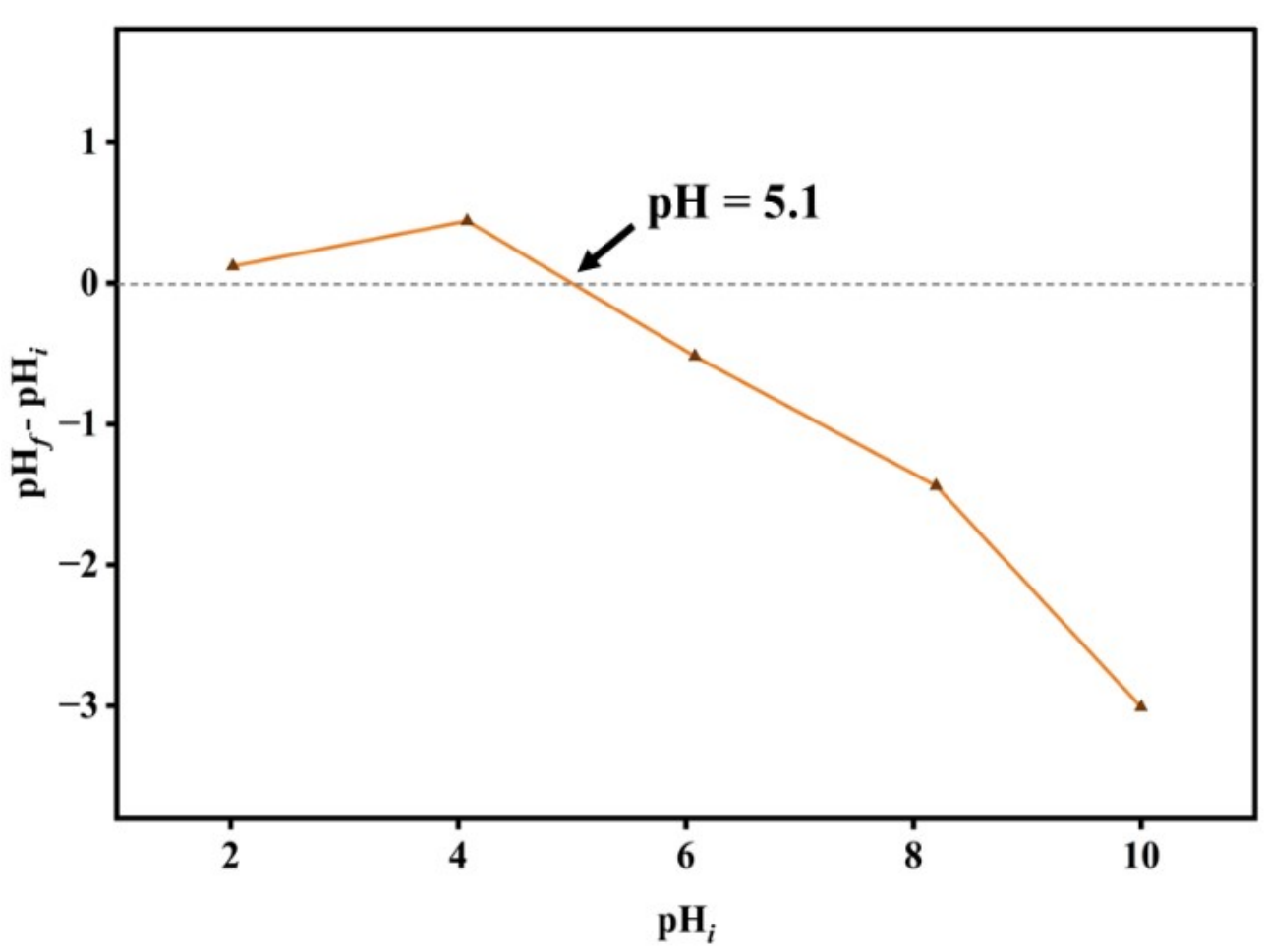

Fig. S4. Determination of the point of zero charge of Ac-NWHHC.

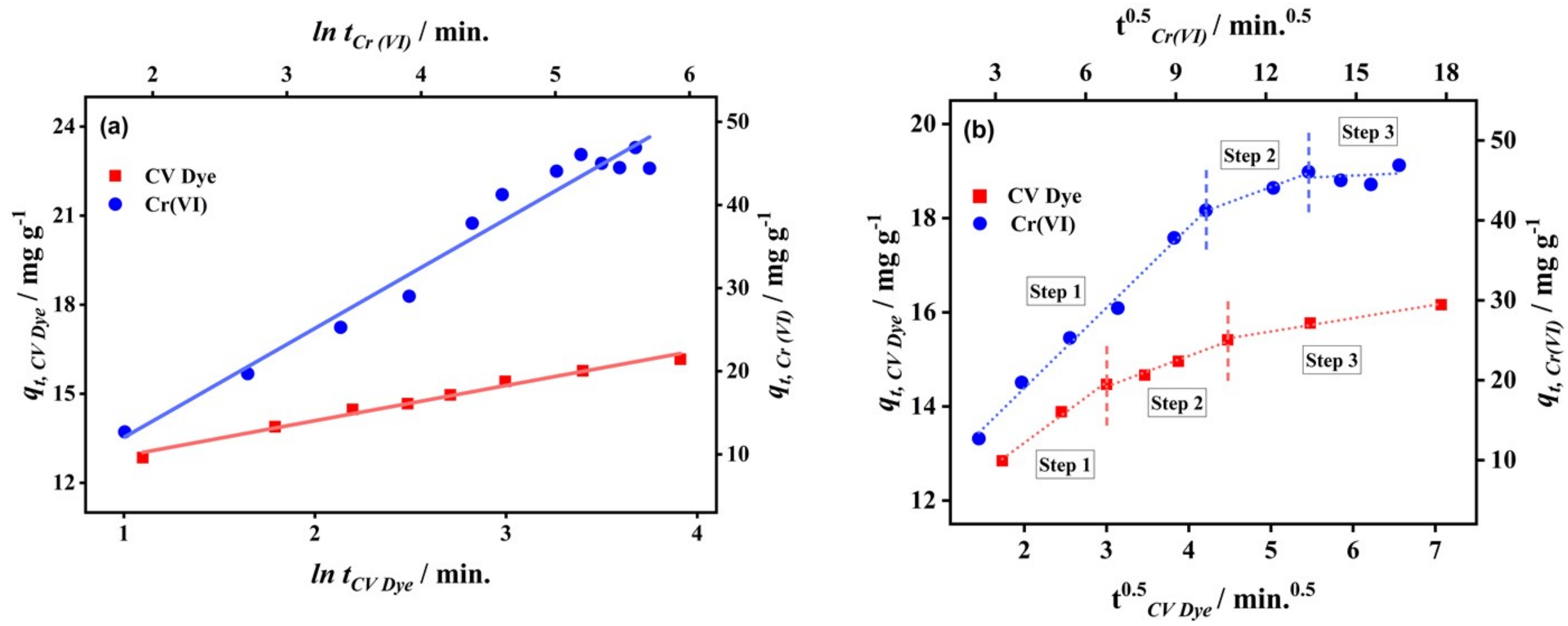

Fig. S5. The linear plots of the (a) Elovich and (b) W-M kinetic models for the adsorption of CV dye and Cr (VI) onto Ac-NWHHC

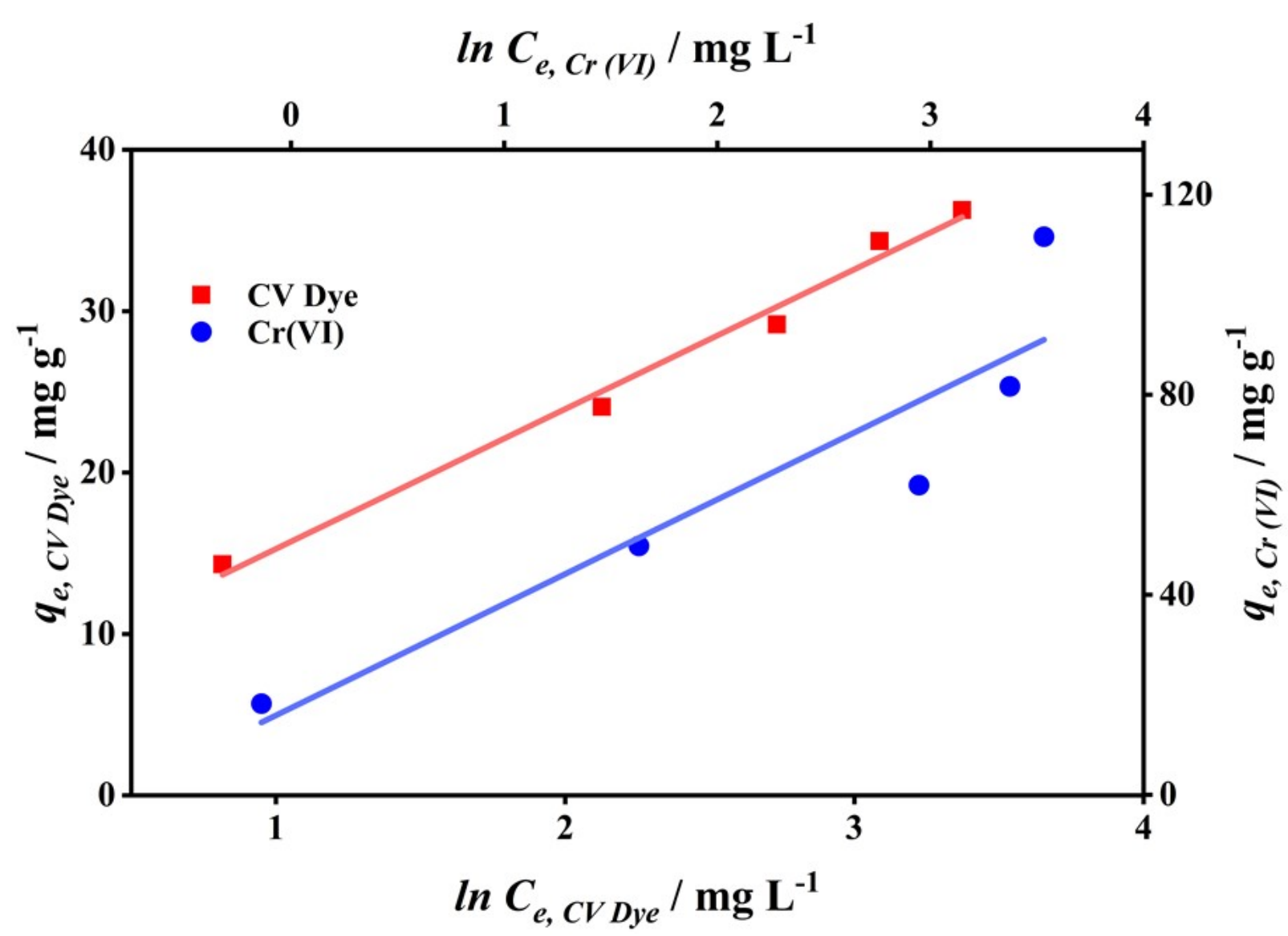

Fig. S6. The linear fitting of the Temkin adsorption isotherm models for the adsorption of CV dye and Cr (VI) onto Ac-NWHHC.

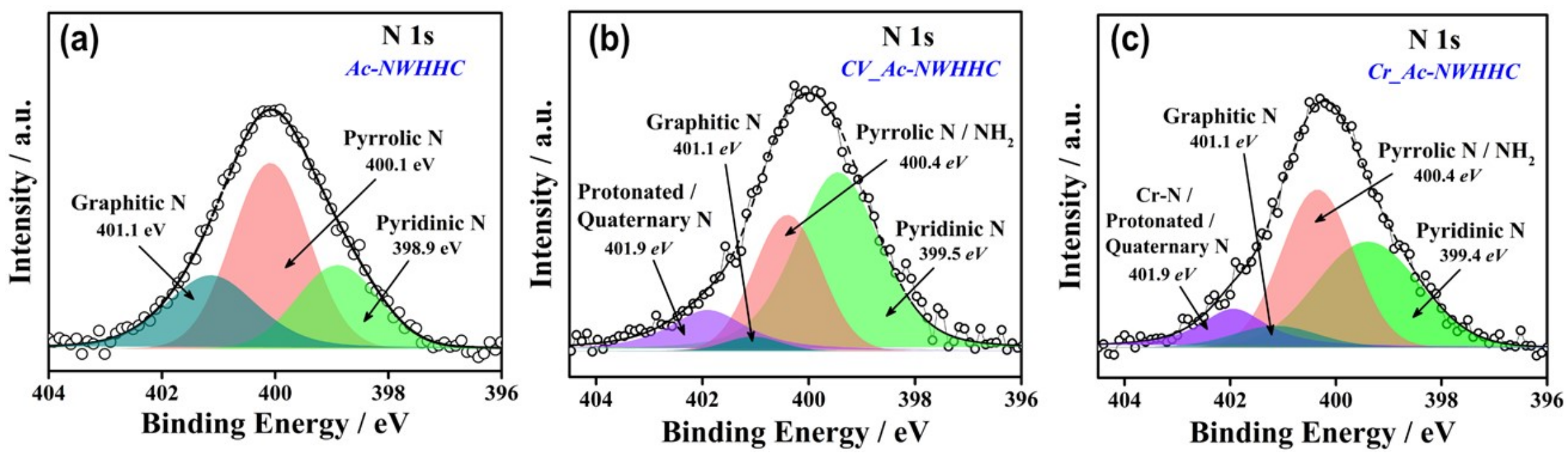

Fig. S7. The N 1s XPS spectra of (a) Ac-NWHHC, (b) CV_Ac-NWHHC, and (c) Cr_Ac-NWHHC.
